# Lost in folding space? Comparing four variants of the thermodynamic model for RNA secondary structure prediction

**DOI:** 10.1186/1471-2105-12-429

**Published:** 2011-11-03

**Authors:** Stefan Janssen, Christian Schudoma, Gerhard Steger, Robert Giegerich

**Affiliations:** 1 Faculty of Technology, Bielefeld University, 33615 Bielefeld, Germany; 2 Bioinformatics Group, Max Planck Institute of Molecular Plant Physiology, 14476 Potsdam, Germany; 3 Institut für Physikalische Biologie, Heinrich-Heine-Universität Düsseldorf, 40204 Düsseldorf, Germany

## Abstract

**Background:**

Many bioinformatics tools for RNA secondary structure analysis are based on a thermodynamic model of RNA folding. They predict a single, "optimal" structure by free energy minimization, they enumerate near-optimal structures, they compute base pair probabilities and dot plots, representative structures of different abstract shapes, or Boltzmann probabilities of structures and shapes. Although all programs refer to the same physical model, they implement it with considerable variation for different tasks, and little is known about the effects of heuristic assumptions and model simplifications used by the programs on the outcome of the analysis.

**Results:**

We extract four different models of the thermodynamic folding space which underlie the programs RNAFOLD, RNASHAPES, and RNASUBOPT. Their differences lie within the details of the energy model and the granularity of the folding space. We implement probabilistic shape analysis for all models, and introduce the *shape probability shift *as a robust measure of model similarity. Using four data sets derived from experimentally solved structures, we provide a quantitative evaluation of the model differences.

**Conclusions:**

We find that search space granularity affects the computed shape probabilities less than the over- or underapproximation of free energy by a simplified energy model. Still, the approximations perform similar enough to implementations of the full model to justify their continued use in settings where computational constraints call for simpler algorithms. On the side, we observe that the rarely used level 2 shapes, which predict the complete arrangement of helices, multiloops, internal loops and bulges, include the "true" shape in a rather small number of predicted high probability shapes. This calls for an investigation of new strategies to extract high probability members from the (very large) level 2 shape space of an RNA sequence. We provide implementations of all four models, written in a declarative style that makes them easy to be modified. Based on our study, future work on thermodynamic RNA folding may make a choice of model based on our empirical data. It can take our implementations as a starting point for further program development.

## Background

### Motivation

A wide variety of bioinformatics tools exist, which help to analyze RNA secondary structure based on an experimentally supported, thermodynamic model of RNA folding [1]. Typical tasks performed by such tools are

• prediction of a single, "optimal" structure of minimal free energy,

• computation of near-optimal structures, either by complete enumeration up to a certain energy threshold, or by sampling from the folding space,

• computation of base pair probabilities and dot plots,

• computation of representative structures of different abstract shapes, or

• computation of Boltzmann probabilities, either of individual structures, or accumulated over all structures of the same abstract shape.

From a macroscopic point of view, all these approaches are based on the same thermodynamic model, but when checking in detail, this does not hold. Algorithms for different tasks make certain assumptions about the folding space, where little is known to which extent these assumptions influence the outcome of the analysis.

The present study is designed to fill this gap. We explicate the details of four different models of the RNA folding space, named NoDangle, OverDangle, MicroState and MacroState. They capture four different models of the folding space, as they are implemented in the programs RNAFOLD[2], RNASHAPES[3], and RNASUBOPT[4].^1 ^We compare the outcome of predictions from the different models, and evaluate them against three data sets derived from experimentally proved structures.

### Goals of the evaluation

The goal of this study is not to define a "correct" or "best" way of modeling the RNA folding space. Different definitions may retain their merits in the light of different computational constraints. We want to explicate the differences in the results which are due to the choice of a particular model. Aside being interesting in its own right, this allows future algorithms designers to make a well-founded choice of the model they base their work on.

How to compare the performance of different models? A first idea would be to evaluate them with respect to prediction of the structure of minimum free energy (MFE; for details see below), using a reference set of trusted structures. This has been done occasionally [1,5], and we will include such an evaluation here for the sake of completeness. However, MFE structure prediction is notorious in the sense that a slight offset in energy can lead to a radically different structure. This is a consequence of the underlying thermodynamic model, and not due to its inadequate implementation. For a more robust evaluation, we need a measure which constitutes a more comprehensive characteristic of the overall folding space of an RNA molecule, including evidence for competing near-optimal structures of significant structural variation.

Abstract shapes of RNA [3,6] provide such a measure. This approach provides two essential types of analysis: (1) to compute a handsome set of representative, near-optimal structures, which are different enough to be of interest, and (2) to compute shape probabilities, which accumulate individual Boltzmann probabilities over all structures of the same shape. The shape probability is a robust measure of structural well-definedness, and in contrast to folding energy, it is independent of base composition and meaningful for comparing foldings of different sequences with similar length.

Types (1) and (2) of abstract shape analysis are achieved by different algorithms, using different models of the folding space, in the program RNASHAPES. A similar situation prevails within the Vienna RNA package, where different models of the folding space are used with various functions of RNAFOLD and RNASUBOPT under different parameter settings.

For our evaluation, we implement probabilistic shape analysis in four different ways, three of which closely correspond to the folding space models implemented for MFE prediction in RNAFOLD^2^, and two of which correspond to the algorithms used in RNASHAPES. This set of programs will allow us to derive observations about the underlying folding space models.

## Methods

In this section, we recall the definitions underlying the thermodynamic model of RNA folding, and then proceed to specify four different implementations of this model.

### The thermodynamic model

#### Free energy and partition function

Structure formation of a single-stranded nucleic acid sequence *x*--from an unfolded, random coil structure *c *into the folded structure *s*--is a standard equilibrium reaction with temperature-dependent free energy $\Delta G_{T}^{0}$ and equilibrium constant *K_T_*:

$$\begin{array}{ccc} c & ⇌s & \\ K_{T} & =\frac{\left[s\right]}{\left[c\right]}\\ \Delta G_{T}^{0} & =-RT\ln K_{T}\;. \end{array}$$

The number of possible secondary structures of a single sequence, i. e. the folding space *F*(*x*) of *x*, grows exponentially with the sequence length *n *[7,8]. These possible structures *s_i _*of a single sequence coexist in solution with concentrations dependent on their free energies Δ*G*^0^(*s_i_*); that is, each structure is present as a fraction $p_{s_{i}}$ according to its Boltzmann probability

$$p_{s_{i}}=\exp\left(-\frac{\Delta G_{T}^{0}\left(s_{i}\right)}{RT}\right)∕Q$$

given by its molar Boltzmann weight $\exp\left(-\Delta G_{T}^{0}\left(s_{i}\right)∕\left(RT\right)\right)$ and the partition function *Q *for the ensemble of all possible structures

$$Q=\underset{\text{all}\;\text{structures }{\text{s}}_{i}\in F\text{(}x\text{)}}{\sum }\exp\left(-\frac{\Delta G_{T}^{0}\left(s_{i}\right)}{RT}\right)\;.$$

The structure of lowest free energy is called the (thermodynamically) optimal structure or structure of minimum free energy (MFE).

RNA secondary structures are conveniently represented as dot-bracket strings, such as

(1) “((.((((..(((...))).....((.((.....))...)).))))))”

where matched parentheses indicate a base pair and dots indicate unpaired bases.

#### Abstract shapes

Many of the possible structures differ from each other by only tiny structural rearrangements like addition or removal of a base pair, or a slight shift in position of a small bulge loop. Structures can be pooled according to their abstract shape. Generally, an abstract shape gives information about the arrangement of structural elements such as helices, but no concrete base pairs [3,6]. The MFE structure within each shape class is called "shrep", which is short for shape representative structure. The partition function *Q_p _*for the ensemble of all structures of shape *p *is

Qp= ∑all structures si∈p exp-ΔGT0(si)RT.

Of course, the structures from all shape classes sum up to the ensemble of all structures:

$$Q=\underset{\text{all shape classes }p}{\sum }Q_{p}$$

and the probability of shape *p *is

$$Prob\left(p\right)=Q_{p}∕Q\;.$$

Shape abstraction can be defined in various ways. RNASHAPES provides shape abstraction functions *π*_1_, ..., *π*_5 _which implement different levels of abstraction, with *π*_5 _being the most abstract. Shapes can be represented as strings, similar to structure representations, where a single pair of square brackets marks a helix (of any length), and an underscore marks a stretch of unpaired bases, also of any length. Levels of abstraction differ in the amount of information they retain about unpaired regions. The above RNA structure (1) is mapped to shape strings on abstraction levels 2 and 5 as follows:

$$\begin{array}{c} \pi_{2}:\;\;\text{“}\left[\text{\_}\left[\left[\right]\left[\text{\_}\left[\right]\text{\_}\right]\right]\right]\text{”}\\ \pi_{5}:\;\;\text{“}\left[\left[\right]\left[\right]\right]\text{”} \end{array}$$

Both shapes indicate that the structure is a so-called Y-shape, a multiloop with a two-way branch. This most abstract view is conveyed by abstraction level 5. The less abstract level 2 shape indicates, in addition, that the outer stem is interrupted by a bulge on the 5' side, and that the 3' branch inside the multiloop is interrupted by an internal loop. For a detailed definition of shape abstraction levels, see [9].

#### Implementing the basic energy model - no dangling bases

In the usual approximation, the free energy of an individual structure *s *is the sum of the energetic contributions of all structural elements of *s*:

$$\Delta G_{T,s}^{0}=\underset{\text{helices}\;j}{\sum }\Delta G_{T,j}^{0}+\underset{\text{loops}\;k}{\sum }\Delta G_{T,k}^{0}$$

with energy of an individual helix:

$$\Delta G_{T,\text{helix}}^{0}=\underset{\begin{array}{c} \text{base pair}\\ \text{stacks }m \end{array}}{\sum }\Delta G_{T,m}^{0}.$$

**Figure 1:**
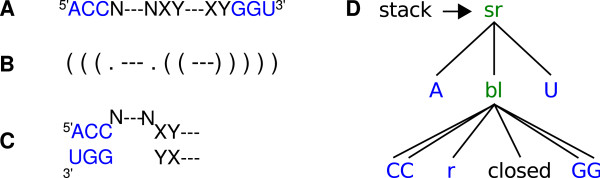
**Example on structure representations**. A sequence, shown in A), folds into a structure that is represented by the three equivalent illustrations in B-D). The structure consists of a helix with three base pairs (ACC paired with GGU), a bulge loop (N--N; N meaning aNy nucleotide), and a helix with two base pairs formed by any complementary nucleotides. The dashes designate omitted sequence stretches. The structure in B) is in dot-bracket notation; that is, dots mark unpaired nucleotides and pairs of opening and closing brackets mark a base pair. The structure in C) is the usual squiggly representation. D) is the tree representation of the same structure: a stacked region (sr) is formed by an A:U pair stacked on top a bulge loop (bl) including two stacking pairs (C:G/C:G) and a loop region with one or more residues (r) on the left (5') side. The helix continues with a "closed" structural element (which is defined as any substructure starting with a base stack).

That is, the energy of a helix depends only on its type of base pairs (G:C, C:G, A:U, U:A, G:U, U:G) stacking on its neighboring base pair [10]. The minimum length of a helix is two base pairs (one base pair stack). Single (lonely) pairs should not exist. The energy of a loop depends on its type (hairpin loop closed by a helix, internal and bulge loop closed by two helices, and multiloop or junction closed by more than two helices), the sequence(s) of loop nucleotides, and type of closing base pair(s). That is, the free energy of a given secondary structure *s *is obtained by decomposition of *s *into its structural elements and summation of values obtained by respective calls of the elementary energy functions of these elements as listed in Table 1. With the example shown in Figure 1, this would be three calls to *sr*_*energy *for the three base pair stacks ($\begin{array}{c} {}^{5^{\prime }}\text{A}{\text{C}}^{3^{\prime }}\\ {}_{3^{\prime }}\text{U}{\text{G}}_{5^{\prime }} \end{array}$, $\begin{array}{c} {}^{5^{\prime }}\text{C}{\text{C}}^{3^{\prime }}\\ {}_{3^{\prime }}\text{G}{\text{G}}_{5^{\prime }} \end{array}$, and $\begin{array}{c} {}^{5^{\prime }}\text{X}{\text{Y}}^{3^{\prime }}\\ {}_{3^{\prime }}\text{Y}{\text{X}}_{5^{\prime }} \end{array}$), a call to *termau*_*energy *for the terminal $\begin{array}{c} {}^{5^{\prime }}\text{A}\\ {3^{\prime }}_{\text{U}} \end{array}$ pair, and a call to *bl*_*energy *for a bulge loop with sequence ^5'^N--N^3' ^and closing pairs $\begin{array}{c} {}^{5^{\prime }}\text{C}\\ {}_{3^{\prime }}\text{G} \end{array}$ and $\begin{array}{c} {}^{5^{\prime }}\text{Y}\\ {}_{3^{\prime }}\text{X} \end{array}$.

**Table 1:** Elementary functions in the basic thermodynamic energy model

Function	Description
*sr*_*energy*	The most important source for stabilizing an RNA secondary structure is stacking of two (or more) base pairs.
*termau*_*energy*	A base pair A:U at the terminal end of a stacking region adds less stabilizing energy than *within *a stacking region.
*hl*_*energy*	Stabilizing contribution for the loop-closing base pair stack plus destabilizing contribution for the hairpin loop region plus bonus energy for special loop sequence (e. g. extrastable tetra loops).
*bl*_*energy*	Analog to *hl*_*energy*, but for a destabilizing loop region bulged out to the left.
*br*_*energy*	Symmetric case to *bl*_*energy*.
*il*_*energy*	Analog to *hl*_*energy*, but with two destabilizing loop regions.
*ml*_*energy*	Since a multiloop of *x *stems is less stable than *x *adjacent stems, it gets a penalty.
*ul*_*energy*	Each stem in a multiloop gets an initial penalty.
*ss*_*energy*	Regions of unpaired bases could get penalized, but we set this value to zero.
*sbase*_*energy*	Same as *ss*_*energy*, but for a single unpaired base.

#### Implementing the full energy model - with dangling bases

In addition to the basic energy model described above, unpaired bases at the end of a helix can stabilize the helix by stacking on the terminal base pair [11-13]^3^.

Introducing dangling bases effectively refines our notion of structure. Any secondary structure, as defined solely by its set of base pairs, can now have several variants according to different choices of dangling bases. Such refinement can be reflected in our structure representation by replacing certain dot symbols by "d", indicating a base dangling onto a helix to its left, and "b" for a base dangling onto a helix to its right. For example, a structure like

“((..((...)).((...)).))”

now has dangle variants such as

$$\begin{array}{c} \text{“}\left(\left(\text{d}.\left(\left(...\right)\right)\text{b}\left(\left(...\right)\right)\text{b}\right)\right)\text{”}\\ \text{“}\left(\left(.\text{b}\left(\left(...\right)\right)\text{b}\left(\left(...\right)\right)\text{b}\right)\right)\text{”}\\ \text{“}\left(\left(\text{db}\left(\left(...\right)\right)\text{b}\left(\left(...\right)\right)\text{b}\right)\right)\text{”}\\ \text{“}\left(\left(..\left(\left(...\right)\right)\text{d}\left(\left(...\right)\right)\text{b}\right)\right)\text{”}\\ \text{“}\left(\left(..\left(\left(...\right)\right)\text{b}\left(\left(...\right)\right).\right)\right)\text{”} \end{array}$$

and 31 more. Each end of a helix can have dangling bases, except an end which leads to the hairpin loop. In this case, energy contributions from dangling bases are already incorporated in the energy parameters for the loops.

Given a concrete secondary structure, it is no problem to consider all possible dangles and compute the optimal energy for this structure. The program RNAEVAL from the Vienna Package can be used for this purpose. However, for structure prediction from a primary RNA sequence, dangle means trouble, as we shall see shortly.

### Modeling folding spaces with tree grammars

#### Tree representation of structures

All approaches using the thermodynamic model are implemented via dynamic programming. Recursively, structures are composed from smaller substructures. Such a dynamic programming algorithm always has an underlying grammar, which describes all the candidates in the folding space of a given RNA sequence. Hence, by extracting the grammars behind different algorithms, we can analyze the differences in their respective folding space in a precise way, and without obscuring implementation detail.

The grammars we use are tree grammars. Non-terminal symbols designate different components of secondary structure, such as a stacking region or a bulge loop. Function symbols in the tree grammar are used to indicate how structures are built up from smaller components. For example, a snippet of a tree structure such as shown in Figure 1 designates at its bottom an unpaired stretch of one or more bases (r), 5' of a *closed *substructure of any type. This situation is indicated by the function symbol **bl**, which stands for "bulge left". The unpaired stretch and the substructure is surrounded by two stacking (C:G) base pairs, and enclosed in yet another base pair, added by function **sr**, which extends a "stacking region". These functions can be seen as actual constructors of a tree-like data structure, representing secondary structures. They can (and will) also be seen as functions, which all call upon the energy functions of the thermodynamic model, to compute either free energies or their corresponding Boltzmann weights. We can also interpret them as functions which count base pairs in the structure they build, or compose the dot-bracket string for that structure, compute their abstract shape, and so on. Modeling structures as trees built from functions that can be interpreted in different ways provides a uniform and flexible formalism for many purposes.

#### From tree grammars to folding algorithms

Tree grammars modeling the folding space of RNA essentially constitute executable code. They can be literally transcribed into a language supporting the algebraic dynamic programming technique [14]. We use the language GAP-L as provided in the recent Bellman's GAP programming system [15,16]. This approach is essential for the study at hand. It takes from us not only the burden to implement and debug dynamic programming recurrences for each of the four algorithms. It also guarantees that the different algorithms correctly implement their respective models, share the energy model, are implemented with the same degree of optimization, and are independent of the programming skills of a bunch of graduate students.

#### Grammars and their relation to established structure prediction programs

We will present four grammars, NoDangle, OverDangle, MicroState and MacroState. The first three implement the folding space of RNAFOLD used with options -d0, -d2, and -d1, respectively. The grammars MicroState and MacroState implement the folding space of RNASHAPES in its two functions. All four grammars will then be empowered with shape abstraction, and are used in our evaluation for computing shape probabilities under the different models.

All grammars use the same energy parameters, but in a different way. The 16 functions of the energy model, as specified in Tables 1 and 2, are used in different combinations by the evaluation functions in the grammars. For example, in all grammars the function *ml *calls the model function *termau*_*energy*, *sr*_*energy*, and *ml*_*energy*. Table 3 provides the cross-references between the energy functions in our programs to be described below, and the energy functions of the thermodynamic model.

**Table 2:** Energy functions for dangling bases

Function	Description
*dl*_*energy*	A single base left of a closed substructure can dangle onto this stack and thus might further stabilize it.
*dr*_*energy*	Symmetric case to *dl*_*energy*.
*ext*_*mismatch*_*energy*	Two bases left and right of a stack, which do not form a basepair (they mismatch), can dangle from both sides to the stack.
*dli*_*energy*	A multiloop is closed by one stack. A single base at the inside of the multiloop and directly next to the closing stack might dangle from left onto this stack. The energy values are the same as *dr*_*energy*, but for a reversed subsequence.
*dlr*_*energy*	Symmetric case to *dli*_*energy*.
*ml*_*mismatch*_*energy*	Two bases on both inner sides of a multiloop closing stack may dangle from inside onto this stack, but do not form a basepair (mismatch).

**Table 3:** Cross-reference between the energy functions in our programs, and which energy contributions (model functions) they call upon.

Function	Used in evaluation function			
	NoDangle	OverDangle	MicroState	MacroState
*termauenergy*	ml	ml	ml	ml
			mldl	mldl
			mldr	mldr
			mldlr	mldlr
				mladl
				mladr
				mladlr
				mldladr
				mladldr
	drem	drem	drem	drem
			edl	edl
			edr	edr
			edlr	edlr

*dl*_*energy*			edl	edl
				ambd
				ambd'
				acomb
				mladl
				mladlr
				mladldr

*dr*_*energy*			edr	edr
				ambd
				ambd'
				acomb
				mladr
				mladlr
				mldladr

*ext_mismatch_energy*		drem	edlr	edlr

*dli*_*energy*			mldl	mldl
				mldladr
				mladl
				mladlr
				mladldr

*dri*_*energy*			mldr	mldr
				mladldr
				mladr
				mladlr
				mldladr

*ml_mismatch_energy*		ml	mldlr	mldlr

*sr*_*energy*	sr	sr	sr	sr
	hl	hl	hl	hl
	bl	bl	bl	bl
	br	br	br	br
	il	il	il	il
	ml	ml	ml	ml
			mldl	mldl
			mldr	mldr
			mldlr	mldlr
				mladldr
				mladr
				mladl
				mladlr
				mldladr

*hl_energy*	hl	hl	hl	hl

*bl_energy*	bl	bl	bl	bl

*br_energy*	br	br	br	br

*il_energy*	il	il	il	il

*ml_energy=3.4*	ml	ml	ml	ml
			mldl	mldl
			mldr	mldr
			mldlr	mldlr
				mladlr
				mldladr
				mladldr
				mladr
				mladl

*ul_energy=0.4*	incl	incl	incl	incl
	ml	ml	ml	ml
				ssadd

*ss_energy=0*	addss	addss	addss	addss
				ssadd

*sbase_energy=0*	sadd	sadd	sadd	sadd

This table shows the use of the very same energy functions for all grammars. Energy differences only stem from different combinations. In the first column, we list the energy model functions. The next four columns contain the evaluation functions of the four grammars.

To retrieve the energy of the example structure of Figure 1 for NoDangle, you should read the table like this: The first evaluation function of the structure is *sr*. Look for all rows in column two where *sr *appears. It is just the case for *sr*_*energy*. Next is *bl*, which again shows up in the row for *sr*_*energy *but also for *bl*_*energy*. The concrete energy values depend on the concrete input bases, thus one should understand the model functions as table look-ups with the bases as parameters. The energy of the whole structure is just the sum of all local energy contributions.

Some evaluation functions do not use model functions. The four variants of the evaluation function *cadd *and *combine *just add energies from their left and right substructures. *Trafo *and *incl *do not change the energy value at all and *nil *simply returns 0.

### Model NoDangle

NoDangle is our grammar incorporating the elementary energy model, without considering dangling bases at all. It corresponds to the model underlying RNAFOLD when used with option -noLP -d0^4^. It is also used in RNASUBOPT. We give a narrative explanation of how this grammar works.

**Figure 2:**
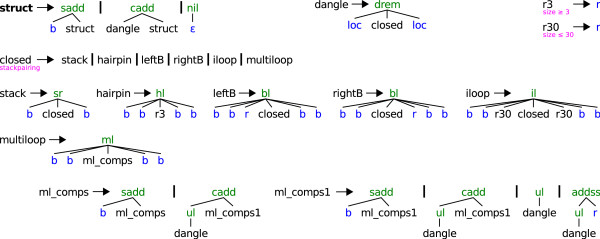
**Grammar for "NoDangle" and "OverDangle"**. The axiom is ***struct***. Alternative productions starting at the same non-terminal are separated by vertical bars. Terminals, b (a single base), r (a region of bases), ε (the empty word) and loc (the position of a neighbored subword), are colored in blue. Green algebra function names, e. g. *sadd *or *hl*, help to write the structures as trees, and are used to associate thermodynamic energies with the structures. Magenta colored words beneath non-terminals are filters, e. g. "stackpairing" requires that the two leftmost bases of the substructure can make base pairs with the two rightmost ones. All different secondary structures for a given RNA sequence, i. e. its complete folding space, can be enumerated by parsing the sequence with grammar NoDangle. The grammar is non-ambiguous in the sense that each structure is found exactly once.

Each complete structure is a ***struct***, i. e. it is derived from the axiom of the grammar (see Figure 2). It might have leading unpaired bases (**sadd**), hold one or more closed substructures (non-terminal *dangle*, function **cadd**), or just end with the empty word (**nil**). A *dangle *is a closed substructure whose directly neighbored bases *might *dangle onto the stack of base pairs. We keep the name *dangle *for consistency with the other grammars, but no dangle energies are considered in NoDangle; the function **drem **simply passes on the energy of its *closed *substructure, which may include a penalty for a terminal A:U pair if appropriate.

A *closed *substructure is a *stack *of base pairs which eventually leads to one of five structural motifs: hairpin loop (*hairpin*), bulge to the left (*leftB*), bulge to the right (*rightB*), internal loop (*iloop*) or *multiloop*. The multiloop is a concatenation (*ml*_*comps *and *ml*_*comps1*) of two or more substructures, embraced by one closing stack. Note that all motifs have at least two closing base pairs which form a stack. This implements the convention of disallowing lonely pairs. The helix initiated by two closing pairs can be elongated by **sr**. A region (r) is a non-empty stretch of unpaired bases (**b**), whose length can be further constrained, e. g. to be at most 30 bases (*r30*) for internal loops or at least 3 bases (*r3*) for a hairpin loop.

The algebra functions **drem **and **ml **control the dangling behavior, which is the only difference between NoDangle and OverDangle. In NoDangle, they do not make any dangling energy contributions at all.

### Model OverDangle

OverDangle is the grammar which considers dangling base energies in a simplified form. It corresponds to RNAFOLD called with options -noLP -d2^5^. The grammar itself is identical to NoDangle (cf. Figure 2). It computes the same folding space, but evaluates energies differently. It assumes an energy contribution from dangling bases on every side of a helix, even if a base is not available for dangling, for example because it is itself engaged in another helix, or already dangling there. The algebra functions **drem **and **ml **control the dangling behavior, which is the only difference between NoDangle and OverDangle. In OverDangle **drem **and **ml **always adds dangling energies for left and right dangles. This is why the production using **drem **uses two *loc *symbols: *loc *recognizes the empty word, and returns its position in the sequence. These positions are used by **drem **to look at the two bases to the left and right of the *closed *substructure.

This "overdangling" model is used because a correct treatment of dangles is much more complicated, as we shall see below. As a plausibility argument in favor of this heuristic, one may say that when a base is overdangled, for example between two adjacent helices, as with the midpoint in "((...)).((...))", this can be seen as a bonus for co-axial stacking of the two helices. Including full co-axial stacking could be considered as a further refinement of the folding space beyond the MicroState model, which will be described below. Still, due to overdangling, the MFE energy value computed may be smaller than actually assigned by the thermodynamic model to the underlying structure. Partition function computations in RNAFOLD use the OverDangle approach, and so does RNASUBOPT with option -d2 (and even -d1, but see below).

Would we use both NoDangle and OverDangle to produce a list of all structures in the folding space, sorted by free energy, these lists would hold the same structures, but in a different order. The true MFE structure (under the full model with correct dangles) will be near the front of each list, but it is not guaranteed to come out on first place. Our next two grammars are designed to achieve this goal.

### Model MicroState

Grammar MicroState is a grammar which refines our model of a secondary structure. It corresponds to RNAFOLD -noLP -d1^6 ^and is used in the 2004 release of RNASHAPES[3] for the computation of representative structures of different shape.

**Figure 3:**
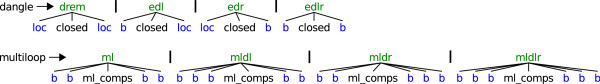
**Grammar MicroState extends the rules of grammars NoDangle or OverDangle for the non-terminal symbols "dangle" and "multiloop"**. Instead of just one way, we now have four alternatives to dangle bases onto a closed substructure: Both neighboring bases do not dangle (*drem *and *ml*), only the left neighbored base dangles onto the stack (*edl *and *mldl*), only the right one (*edr *and *mldr*), or both ones (*edlr *and *mldlr*).

MicroState has separate rules for a helix end with two bases, one base or no base dangling onto it (see Figure 3). These four cases compete with each other for minimum free energy. If surrounding bases are already base paired, only the **drem **case applies (no dangles). If it is decided (say) that the left neighboring base dangles onto the helix, then this base is not available for also dangling on another helix. In this way, grammar MicroState correctly finds the structure of minimal free energy, and could, in principle, also explicitly report the optimal dangles, as in "..b((...))d((...))...".

All variants of the same secondary structure, augmented with different dangles, are now separate members of the folding space. In contrast to the classical model, accounting only for base pairs, we call them "microstates". Let us derive a rough estimate of this folding space enlargement. The size of the folding space for a sequence of length *n *grows asymptotically with *a *· *b^n ^*· *n *^-3/2^, with *b *= 1.44358 and *a *= 3.45373 [8]. A structure has, on average, *k*(*n*) helices, where *k *grows with *n*. Each helix end has up to four ways to play with the dangles, but helix ends in hairpin loops do not count. Directly adjacent helices further reduce the number of dangling alternatives.

**Figure 4:**
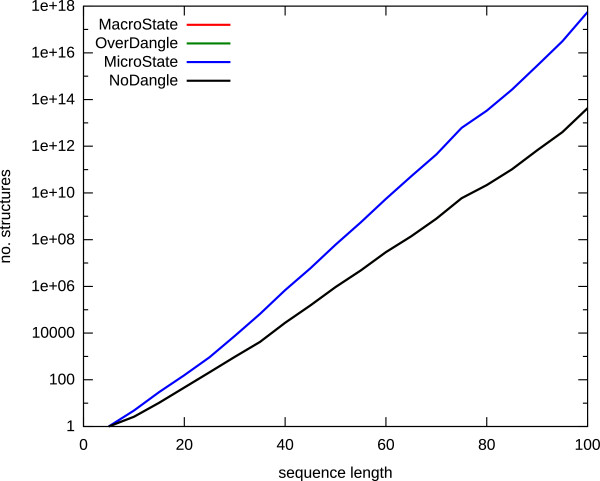
**Growth of folding spaces for all four grammars**. We used uniformly distributed random sequences, with step-size 5 bp. The number of secondary structures heavily depends on sequence composition, thus we took the average over 100 sequences per data point. Curves for "MacroState" and "OverDangle" are not visible, because they are perfectly overlayed by "NoDangle", i. e. all three folding spaces have exactly the same size.

Let us, for simplicity, assume that an helix has 4 dangle variants on average. Then, the above formula changes for the number of microstates to *a *· 4^*k*(*n*) ^· *b^n ^*· *n *^-3/2^. An empirical measurement is shown in Figure 4. From the measurements, and for their particular data sequences and lengths, we can estimate $k\left(n\right)\approx \frac{n}{15}$. For a sequence of length 100, for example, we see an increase by a factor of 10^4^. Clearly, this is a substantial enlargement of the folding space, and different structures are affected to a different extent. (For example, the open structure (no base pairs) gives rise to only one microstate.)

This enlargement of the search space is not a problem for MFE structure prediction. The dynamic programming algorithm derived from the grammar MicroState only does a constant amount of extra work compared to NoDangle and OverDangle. But a severe problem arises with the desire to investigate near-optimal structures. The roughly 4*^k ^*microstates of an optimal structure with *k *helices crowd the near-optimal folding space, while representing the same structure in the non-dangling sense. Enumerating suboptimals returns a tremendous amount of useless information. RNASUBOPT therefore uses OverDangle for enumeration, even when option -d1 is specified. Afterwards, it re-evaluates the energy of predicted structures using correct dangling. Hence, the ranking of structures may change. Occasionally, we observe that the energy of the true MFE structure is so much above the energy of other, overdangled structures that it falls above the energy threshold for enumeration and is not returned at all.^7^

The second problem arises with computations that are based on Boltzmann statistics. The partition function *Q *sums up the Boltzmann-weighted energies of all members in the folding space. Each secondary structure contributes to the partition function as many times as it has microstates, hence the result would be skewed towards structures with many microstates. The significance of this bias is hard to judge^8^, and up to this study, it could not be evaluated empirically. For this reason, RNAFOLD does not support partition function computation with the MicroState model (option -d1).

Fortunately, the partition function with correct dangles, avoiding overdangling as well as explosion of the folding space, can also be computed. To keep the folding space simple, we need a more sophisticated grammar: MacroState.

### Model MacroState

**Figure 5:**
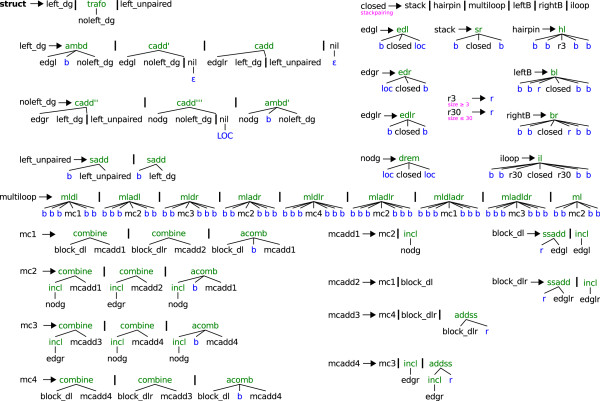
**"MacroState" grammar**. The color code is identical to Figure 2. The basic structure of the "MacroState" grammar is inherited from the previous three grammars, but it has a more complex distinction of cases for dangling bases. "MacroState" has to consider all the different dangling situations as in "MicroState", but its search space is restricted to the *k*(*n*)-times smaller folding space of the input sequence. To achieve these contradicting goals, dangling alternatives do not exist as search space candidates but are implicitly examined within the evaluation algebra. The grammar has to ensure that a substructure is of a defined dangling type whenever its energy or partition function value is used in an algebra evaluation function. We know that any helix derivated from *nodg *has no unpaired bases to its left or right, while helices from *edgl*, *edgr *or *edglr *have exactly one unpaired base dangling from left, right or exactly two unpaired bases dangling from both sides, respectively. In all four cases, there is no unpaired base left for a further dangling. Care must be taken, where we can not be sure if e. g. the leftmost unpaired base of a *block*_*dl *derivation is free to dangle to some helix to its left. The unpaired base would be available for a dangling if we use *ssadd*, but is occupied in *incl *situations. This uncertainty is passed to every calling function, but with a clever grammar design we can at least ensure that its type does not change. For example every *mc1 *or *mcadd2 *derivation contains one or more helices with one or more unpaired bases at its 5' end and definitely no unpaired base at its 3' end. Furthermore *mc2 *and *mcadd1 *always have no unpaired bases to both sides, *mc3 *or *mcadd4 *have one or more unpaired bases only at its 3' end and finally *mc4 *or *mcadd3 *are known to have one or more unpaired bases to both ends. The benefit of these distinctions can be demonstrated with the multiloop functions *mldl *and *mladl*. The important base is the one that is directly left to the *mc1 *or *mc2 *substructure. In principle, it can either dangle to the left, that is the closing stem of the multiloop, or the right, that is the leftmost helix within the multiloop. Actually, for *mldl *our base of interest can only dangle to the left, because every *mc1 *derivation already has at least one further base in front of the first inner helix. For *mladl *we truly have an **a**mbiguous situation, where the base of interest could dangle to one of both sides. Please note that *mldl *and *mladl *correspond to two different dot-bracket structures. *mldl *handles macrostates of the type "((..." including microstates "((..." and "((d..", whereas *mladl *handles macrostates of type "((.((..." and includes the microstates "((.((...", "((d((...", and "((b((...". The mfe algebra function locally chooses the variant with the better free energy, even if a global analysis would reveal that the locally worse structure would become MFE in the end. This constitutes a rare case where the MFE structure may be missed. Our partition function algebra correctly keeps track of these situations.

Grammar MacroState (see Figure 5) follows the overall pattern of the other grammars, but is much more refined. This grammar was designed originally with the 2006 release of RNASHAPES[6] to compute complete probabilistic shape analysis. Its rules are written to record and distinguish the situation where a helix (1) ends with a base pair, (2) already has a single unpaired base to its right or left, or (3) has several unpaired bases on either side. No dangle energies are added in cases (1) and (3), and in case (2), all possible dangle variants (up to four microstates) are evaluated and minimized over while considering the corresponding macrostate. This leads to a much larger number of non-terminal symbols and functions in the grammar. MacroState has 25 non-terminal symbols and 32 functions, compared to NoDangle with 11 non-terminals and 12 functions.

The important feature of MacroState is that for any sequence, it defines the identical folding space as NoDangle. This is hard to believe when just looking at the grammar, but has been shown in [6], and is further demonstrated by the measurements shown in Figure 4. The size of the folding space, as defined by MacroState, agrees with that of NoDangle and OverDangle not only on average, but also on each individual sequence.

What is the effect of using either MicroState or MacroState? Does it really matter? Table 4 shows an extreme example of how the choice of the state space affects the computed probabilities:

**Table 4:** Extreme probability shift example

GACCAAAGCCUUUGUCCCACAAAUUGCGAUCGCGUCGCGGAGC		
**MacroState prob**.	**MicroState prob**.	shape class
58.44%	32.58%	[][]
29.32%	63.43%	[[][]]
12.24%	03.99%	[]

In this example, 40% of the probability mass is shifted by switching models, causing the order of the two top-ranking shapes to be reversed. To find out whether this situation is the exception or the rule is a main motivation of this study.

## Results & Discussion

### Data sets

The four data sets used in this study, DARTS, FR3D:3A, FR3D:4A, and RNAstrand:91 are based on RNA 3D structure data sets prepared in the context of previously published studies.

#### Structures drawn from PDB

We examined three datasets - DARTS, FR3D:3A, and FR3D:4A- based on RNA 3D structural data sets prepared in the context of previously published studies. All three original data sets were created in order to reflect the currently available structural repertoire of RNA molecules as given by structures solved experimentally by X-ray and NMR analysis.

The DARTS set was used for the analysis and classification of RNA tertiary structures in [17]. It was built from all structures available in the March 2007 version of the Protein Data Bank (PDB) [18,19]. The DARTS data set is available at http://bioinfo3d.cs.tau.ac.il/DARTS and contains 244 structures. The creation of this data set involved dedicated structural comparisons to ensure pairwise structural and sequence variability. Unfortunately, the DARTS database is not updated anymore and therefore is limited to data deposited in the PDB before March 2007.

The two FR3D data sets [20,21] are representative sets based on all RNA X-ray structures with a resolution of up to 3 Å (246 structures containing 653 chains) and up to 4 Å (293 structures containing 764 chains), respectively, that were contained in the PDB in 2010. Both sets contain one representative structure for each group of RNA structures found similar (or identical) according to the employed sequence (> 95% identity) and structural (cf. [21]) similarity cutoffs. Both data sets FR3D:3A and FR3D:4A are available as weekly updated lists at http://rna.bgsu.edu/FR3D. The FR3D data sets were created taking recently solved structures into consideration and therefore represent the currently known RNA 3D structural space. Here, the FR3D:3A set is restricted to structures that have been solved at a better resolution and may therefore be more reliable than structures contained in the FR3D:4A set. In turn, the FR3D:4A set has a less strict resolution cutoff and therefore contains more structures.

#### From PDB structures to "gold" structures

In order to generate the data sets for this study, we downloaded all 3D structures contained in the original data sets from the PDB and extracted the secondary structures of each RNA chain using the stereo-geometrical information encoded within the atomic coordinates. Each chain was processed with the base pair annotation software tool MC-Annotate [22] resulting in a list of all intramolecular contacts in the chain. For this study, we only used base pair interactions that are formally involved in secondary structure formation, namely the *cis *Watson-Crick (cWC) base pairs (G:C, C:G, A:U, U:A, G:U, U:G). All other interactions, such as non-canonical base pairs, base stackings, and base-backbone interactions were ignored since they are not part of the secondary structure. The secondary structure of an RNA chain could then be reconstructed directly from the ordered list of canonical base pairs. In a next step, this "preliminary" structure was scanned for lonely base pairs and pseudoknot interactions. Since lonely base pairs are thermodynamically unstable in a secondary structure, they were removed from the list. Due to the fact that there is no unique solution to remove the knot(s) from a pseudoknotted structure, these structures are unusable for the purpose of our study. Therefore, structures containing pseudoknots larger than one base pair, were also discarded. We consider the set of structures reduced in this way as the set of "gold" structures. They constitute our standard of truth, but we are reluctant to call them "true" structures, not only because of our removal of information, but also since structures *in cristallo *may be different from structures *in vivo*^9^.

Our gold data sets resulting from DARTS, FR3D:3A, and FR3D:4A consist of 147, 111, and 136 structures, respectively.

As a final detail: in a few cases, FR3D:3A and FR3D:4A contain the same sequence, with different resolution in 3D and with *different secondary structure *derived from it. No secondary structure prediction program can be expected to be correct in both cases.

##### A data set derived from RNAstrand

Aside from these data sets, we also created a data set RNAstrand:91 with 91 structures from the RNAstrand database [23]. Since RNAstrand was designed as a source of validated structures, with an eye on the evaluation of RNA-related bioinformatics tools, it will be interesting to observe if the findings on this data set agree with the others.

Overall, we shall find that our four data sets deliver consistent sets of results. Therefore, the text of this article will discuss only selected measurements in detail, with the other ones given in the additional file 1, as well as all four raw data sets in additional file 2.

### Evaluation of models for MFE structure prediction

While our main interest is in the effect of the chosen model on the partition function based computations, we here evaluate the four grammars with respect to prediction of a single MFE structure.

#### Evaluation setup

In evaluating models with respect to MFE structure prediction, we include not only our programs NoDangle and OverDangle, MicroState and MacroState, but also the folding programs UNAFOLD and RNAFOLD, which our readers are rightfully curious about because of their practical importance. *Turner'99 *parameters [1] were used throughout^10^. These parameters are derived from melting experiments, with a few exceptions. Multiloop parameters such as *ml*_*energy *in *Turner'99 *are not derived from experiment, but are optimized from structure data to be used in conjunction with the MicroState model. Out of competition, we also include CENTROIDFOLD, which goes beyond strict energy minimization by producing a near-optimal ensemble of structures and choosing the eventual, single-structure prediction based on this sample.

Relative performance of programs of different origin is, however, not our main interest here. Mainly, the evaluation should support that our four grammars faithfully reproduce the behavior of the models underlying RNAFOLD with options -d0, -d1, and -d2, as postulated at the outset of this study.

**Figure 6:**
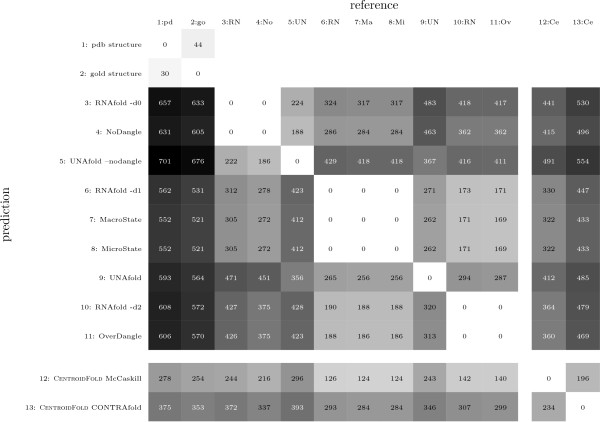
**Comparison of different MFE prediction programs**. **Dataset: **we use the 147 sequences from the DARTS set, except pdb1ajt1B, pdb1kod1A, pdb1koc1A, pdb1lpw1B and pdb1t4x1B, which crashed under UNAFOLD. Together, all according "PDB" structures contain 1,614 base pairs. All "gold" structures have 1,593 base pairs. **Distance: **One base pair set, i.e. secondary structure, is the reference (*R*: table columns), the other one is the prediction (*P*: table rows). Traditional base pair distance is defined as|*R *\*P*| + |*P *\*R*|. Following [34], we decide to allow additional base pairs in the prediction, as long as they are compatible with the reference, i.e. both bases are unpaired and the additional base pair does not introduce a pseudoknot in the reference. The set of compatible base pairs is *P*^-*c *^= *P*\{(*a*, *b*)|(*a*, *b*) ∉ *R *Λ (*a*, *b*) compatible to *R*}. Then, our asymmetric base pair distance is: |*R *\*P*| + |*P*^-*c *^\*R*|. Table values are the sums of base pair distances for all 142 sequences. In the case of co-optimal results, the one with the smallest distance to the reference is chosen. Our distance function is rather strict and does not allow base pair slippage. If a gold base pair (*i*, *j*) is mispredicted as (*i *+ 1, *j*), this contributes a distance of 2. **Programs: **for each RNA sequence we called the programs with the following command line options: RNAFOLD (version 1.8.5): echo sequence | RNAfold -noPS -noLP -dX, where X is 0, 1 or 2. UNAFOLD (version 3.8): hybrid-ss-min --suffix = DAT --mfold --NA=RNA --tmin = 37 --tinc = 1 --tmax = 37 --sodium = 1 --magnesium = 0 --noisolate --nodangle tmpseqfile >/dev/null && ct2b.pl tmpseqfile.ct, with and without the --nodangle switch, where "tmpseqfile" is a fasta file containing the sequence and "ct2b.pl" is a small Perl script from the Vienna Package, which converts RNA structures from "connect" to "dot-bracket" format. CENTROIDFOLD (version v0.0.9): centroid_fold --engine=X tmpseqfile, where *X *is the source of base pair probabilities and is either computed by RNAFOLD (McCaskill) or by CONTRAFOLD. Our ADP implementation of the four grammars "NoDangle", "OverDangle", "MicroState" and "MacroState" get the sequence as their sole input. The binaries can be built with the source code from the additional file 3 and the Bellman's GAP compiler.

The data set in this evaluation is DARTS. Evaluation results are summarized in Figure 6. We use an asymmetric base pair distance for comparison, as explained with Figure 6, where one structure (row entry) is treated as the prediction, the other as the reference (column entry).

#### Observations from MFE prediction experiment

##### Consistency of implementations

Naturally, comparing the results from the same tool leads to entries of zero base pair distance in the diagonal of Figure 6. The off-diagonal zero entries, however, are quite remarkable. When two different algorithms perfectly agree in their MFE predictions on the complete data set, this provides strong evidence that they both faithfully implement the same thermodynamic model of the folding space in each of its variants. In particular, this shows that our grammars NoDangle/OverDangle and MicroState indeed capture the analysis computed by RNAFOLD with options -d0/d2 and -d1. The perfect zeroes might even make our reader suspicious! Occasionally, there must be two (or more) co-optimal structures of minimal free energy, and it is not formally defined which one a program should return in this situation. Hence, it is accidental whether or not two different programs, implemented by different programmers, make the same choice. We therefore have designed our new programs to report all co-optimal solutions in such a situation, and then choose the structure closest to the RNAFOLD prediction. This always delivered a perfect match.

We apply the same technique of safe-guarding against co-optimals when comparing to a database structure. Note that in practice, when predicting structure for a novel RNA, the users of a structure prediction program have no reference structure to resort to. In this case, reporting all co-optimal structures makes them aware of the ambiguity of the situation, and leaves them with the choice to make. This is somewhat preferable to quietly reporting a single MFE structure, selected from several by implementation peculiarities.

The perfect agreement of MacroState with the MFE prediction of RNAFOLD -d1 as well as with MicroState demonstrates that MacroState in fact computes the energy model of the other two programs, while avoiding (as explained above) their explosion of the state space. Taken together, these consistency results shows that we have correct programs set up for our second experiment, where we will evaluate the effect of the chosen energy and state space model on partition function calculations.

##### Quality of MFE predictions

Overall, the quality of MFE predictions compared to "real" structures is moderate when measured on the individual base pair level, with errors^11 ^ranging from 16% to 21% for the gold structures. This is expected and well-known. It is the reason why researchers have developed more advanced techniques, such as structure sampling, complete enumeration, or shape abstraction. The PDB structures contain base pairs which by definition are not predicted - non-standard pairs, 3D interactions, pseudoknots, and lonely pairs. As explained above, the data set of gold structures has been cleaned up in these respects, and as expected, the predictions come closer, but deviations are still considerable.

The gold structures are best predicted by MacroState and MicroState (distance 521) and RNAFOLD -d1 (distance 531). The small difference is accidental and arises from the rare case where RNAFOLD picks an unlucky choice from several co-optimal structures.

##### Performance of different dangling models

Comparing the full dangling model (MicroState, MacroState) to its upper and lower approximations NoDangle and OverDangle, we find that its proper implementation pays off. It reduces the accumulated distance by about 14% over NoDangle, and by 9% over OverDangle. Similar percentages apply for RNAFOLD option -d1 versus -d0 and -d2. This also shows that OverDangle approximates the correct model better than NoDangle and justifies its use as a substitute for the full model in partition function calculations with RNAFOLD and RNASUBOPT, where the grammar MacroState is not available.

##### unafold performance

The two versions of UNAFOLD consistently score a bit worse against the gold structures than all other programs. Compared to each other, we also observe that the distance is improved by considering dangling energies, here by 17%. Otherwise, the two UNAFOLD versions cluster with the NoDangle/MicroState groups, as they should^12^.

##### Looking deeper into the near-optimal folding space

We included CENTROIDFOLD[24] as a representative of methods which, in contrast to the above programs, look deeper into the Boltzmann ensemble of near-optimal structures. Our evaluation shows that the extra effort is well spent. CENTROIDFOLD comes closest to the good structures, and with respect to the single structure predictors, it corresponds best with the group of RNAFOLD -d1, MacroState and MicroState.

### Evaluating models for partition function and related computations

We will explain our evaluations in detail based on our largest data set, DARTS. Results on the other data sets are obtained in an analogous way and are summarized in the end of this section.

#### Evaluation Criteria

In this section, we apply probabilistic shape analysis to our data set. We are interested in the difference of performance of the four models NoDangle, OverDangle, MicroState and MacroState. For simplicity, we call the abstract shape of the reference structure the "reference shape", and refer to the most likely predicted shape as the "dominant shape", although its actual dominance within the Boltzmann ensemble will not be strong if there is another shape with similar probability. The shape string of the reference shape of sequence *s *is obtained by a call to RNAshapes -t l -D "*s*", where 1 is one of the five shape abstraction levels.

We ask the following questions:

• What are the differences in the shape probabilities computed with each of the four models?

• How is the difference affected by the shape abstraction level considered?

Since we do observe significant differences in model behavior, we also ask which model comes closer to the truth:

• To what extend does the dominant shape agree with the reference shape?

• What is the median (or the 75% and 90% quantile) of the reference shape among the predicted shapes?

Finally, we consider

• What are the runtime or memory trade-offs for computing with different models?

##### Evaluation method

Shape probabilities do not make a structure prediction per se. They provide holistic information by assigning probabilities to all shapes in the folding space of a sequence *x*. It is our responsibility how we interpret theses data. The hope is, of course, to find the biologically functional structure among the high-probability shapes, to find two high probability shapes for a riboswitch, to use lack of any shape with high probability as an indicator of absence of a well-defined structure, and so on. Such analysis goes beyond shape probabilities, and takes into account the concrete shreps returned for each shape.

Independent of what the shape probabilities will be used for, we want to focus on the agreement between the four grammars. To measure this, we use the *shape probability shift *(SPS). For a given sequence *x*, all grammars will report the same shape classes, but with different probabilities. Let *P *(*x*) be the *shape space*, i. e. the set of all shape classes for *x*, and *Prob_G_*(*p*) the shape probability of *p *under grammar *G*. The shape probability shift for *x *and grammars *A *and *B *is defined as:

(2) $$SPS_{A,B}\left(x\right)=\frac{1}{2}\cdot \underset{p\in P\left(x\right)}{\sum }|Prob_{A}\left(p\right)-Prob_{B}\left(p\right)|$$

Note that 0 ≤ *SPS*(*x*) ≤ 1, where the extreme case of 1 would only be achieved when all shapes with positive probability by grammar *A *have zero probability by grammar *B *and vice versa. The SPS can be interpreted as the overall probability mass that moves between shapes.

We chose the SPS measure because of this nice interpretation. We also evaluated two alternative measures. The squared distance of base pair probability matrices is correlated with the SPS by a factor around 0.83 at shape level 5 and not much lower on less abstract shape levels. The Kullback-Leibler divergence turned out to be unsuitable for the purpose, as it is not symmetric and both versions (KL(x, y) versus KL(y, x)) show the poorest correlation among all methods tested. Details of this investigation of alternatives are given in additional file 1.

##### Observations

**Figure 7:**
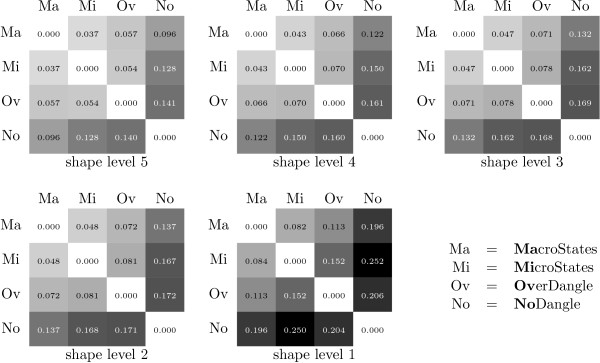
**Model similarity: shape probability shift**.

The values in Figure 7 are average SPS^13 ^over all *x *∈ DARTS, which is the largest of our data sets.

First, consider shape abstraction level 5. We find that models MacroState and MicroState show the most agreement, where the SPS is around 3.7%. MacroState shows a significant SPS against the others, strongest against NoDangle (9.6%) but also against OverDangle (5.7%). A SPS in this range means that while in many cases, the predicted dominant shape will be the same for all models, this need not hold in general.

This justifies the question which of the model finds the gold shape as the dominant shape more often (see below). By the way: the dominant shape and the shape of the MFE structure agree for MacroState in 143 out of 147 cases.

**Figure 8:**
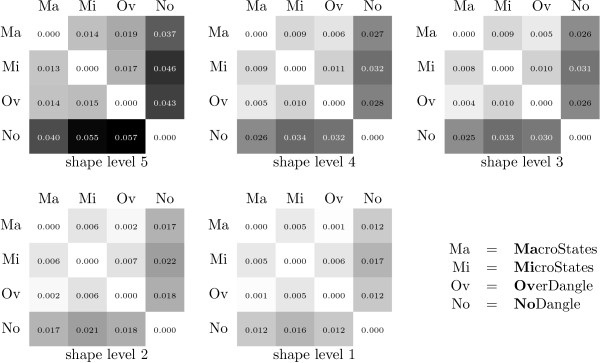
**Model similarity: average shape probability shift per shape**.

Let us next turn from level 5 to decreasinging levels of abstraction. Moving to abstraction levels 4, 3, 2, and 1, the number of shapes increases with each step, while each shape class holds a smaller number of structures. The overall relationship between the models on levels 4 through 1 is consistent with what we observe for level 5. Overall, the SPS values increase. A closer inspection of the raw data shows that SPS values actually decrease for each individual shape, but due to the larger number of (smaller) shifts, their sum increases. Evidence is provided in Figure 8.

##### Dominant shape is gold shape?

The values in Table 5 show the ratios of correct shape predictions vs. the size of the testset, which is 147 in the case of DARTS. We observe the following:

**Table 5:** Ratio of agreement between dominant shape and gold shape for the different grammars (columns) and different shape abstraction levels (rows).

Level	MacroState	MicroState	OverDangle	NoDangle
**5**	0.823	0.816	0.796	0.810
**4**	0.694	0.694	0.660	0.687
**3**	0.687	0.680	0.660	0.673
**2**	0.653	0.653	0.612	0.646
**1**	0.585	0.551	0.565	0.592

The best ratio of agreement of dominant shape and gold shape is 82.3%. The fact that this value is not higher is the reason which makes investigators look into several high-probability shapes and their shreps in practice. Comparing the models, we find that there is no clear winner, with a margin of only 2.7% between the best and the worst performer. (Moreover, the first position varies over our data sets.) Here, MacroState finds agreement most often, with a 0.7% margin over MicroState and 1.3% margin over NoDangle. OverDangle performs worst (79.6%), but not hopeless when we consider that one will look at a number of top-ranking shapes anyway.

Thus, the more interesting question is how the gold shape is placed among the predicted shapes - cf. Table 6. We investigate this aspect by compiling a list of *rank*(*p*^gold^) for all 147 testsequences, sorting this list ascendingly and report the median (50%), the 75%, and the 90% quantile of the list, as well as the complete list (100%). For example, the value 2 for MacroState in shape abstraction level 5 in the 90% column means that, if we decide to take only the top two shapes for closer study, the gold shape is among them in 90% of the cases. Three top shapes are suffice to reach this coverage with MicroState and OverDangle. Overall, the advantage of MacroState appears marginal over the other grammars on level 5, and appears somewhat randomized for weaker abstraction levels.

**Table 6:** Positions of correct shapes.

Level	MacroState				MicroState				OverDangle				NoDangle			
	50%	75%	90%	100%	50%	75%	90%	100%	50%	75%	90%	100%	50%	75%	90%	100%
**5**	2	2	2	8	1	2	3	12	1	2	3	10	2	2	2	4
**4**	1	2	4	85	1	2	4	124	1	2	4	217	1	2	4	64
**3**	1	2	4	108	1	2	6	192	1	2	5	315	1	2	5	54
**2**	1	2	16	759	1	3	21	3729	1	3	13	2404	1	3	21	6534
**1**	1	4	46	1373	1	4	68	6395	1	3	58	2349	1	5	42	4674

An unexpected observation is the strong performance of shape level 2. Considering the 75% quartile, 3 shapes suffice to find the gold shape, independent of the model chosen. We will return to this observation in the Conclusion.

##### Relative runtime and memory consumption

Using the Unix tool "memtime", we logged the "resident set size" as an estimate for memory consumption, see Table 7, and the sum of "user-space" plus "kernel-space" times as an estimate for the process runtime, Table 8, for all test sequences and summed them up for runtime and used the maximum for memory. Since the actual values highly depend on hardware and software issues, e. g. 64 vs. 32 bit or compiler optimizations, we set the MacroState level 5 value (first row, first column) to 1.0 and give all other values relative to it.

**Table 7:** Relative memory.

Level	MacroState	MicroState	OverDangle	NoDangle
**5**	1.00	0.26	0.26	0.21
**4**	3.90	0.76	0.76	0.53
**3**	6.62	1.31	1.24	0.74
**2**	139.12	6.93	7.89	7.36
**1**	795.14	47.38	51.21	24.29

The MacroState level 5 value equals 31.8 MB resident set size.

**Table 8:** Relative runtime.

Level	MacroState	MicroState	OverDangle	NoDangle
**5**	1.00	0.25	0.15	0.12
**4**	3.70	0.95	0.59	0.39
**3**	5.99	1.59	0.96	0.60
**2**	145.56	14.46	9.39	8.37
**1**	643.20	117.16	51.76	28.99

The MacroState level 5 value equals 20.76 seconds on an Intel^® ^Xeon^® ^CPU L5420 @ 2.50 GHz.

MacroState is the most sophisticated grammar and hence the most expensive to compute with. It is slower compared to MicroState, OverDangle, and NoDangle by factors of about 4.0, 6.7, and 8.3, respectively, on level 5. This slowdown factors are about the same for level 4 and 3, and increases for levels 2 and 1, but not consistently so. The largest slowdown measured is 643.20/28.99 = 22.2.

In terms of memory requirements, similar observations hold. This is clear, since all algorithms are implemented via dynamic programming, where a difference in the number of tables to be filled (with MacroState needing the most) directly maps to the difference in runtime as well as in space requirements.

Overall, the selected shape abstraction level makes more difference with resource requirements than the chosen model. For example, NoDangle (the most efficient) used with abstraction level 2 uses more time and space than MacroState (the least efficient) with abstraction levels 5, or 4.

#### Consistent results on data sets DARTS, FR3D:3A, FR3D:4A, and RNAstrand:91

We performed the same analysis as described above for the data set DARTS also for the data sets FR3D:3A and FR3D:4A and RNAstrand:91. Our observations on these data sets are consistent with what was reported above. Therefore, measurement results on these data sets are reported in additional file 1, but not further discussed here.

RNAstrand:91 performing similar to the PDB-derived data sets demonstrates that the RNAstrand data base meets its design goal to provide a solid base of validated structures for tool evaluation [23]. Structures from RNAstrand can be selected according to specifc criteria of interest, and do not require the clean-up operations we had to perform with structures taken from PDB.

## Conclusion

### Model comparison

Summing up our observations from model comparison and model performance evaluation, we conclude the following:

**Conclusion 1 ***For prediction of a single structure, there is no better alternative (among the models considered) than *RNAFOLD -*d1*, *possibly augmented to report ALL structures with the optimal MFE value as in MicroState, when several exist*.

However, with such augmentation, a filter must be provided to safeguard against co-optimal microstates of the same optimal macrostate being reported.

**Conclusion 2 ***The distortion of shape probabilities caused by state space explosion (MacroState versus MicroState) is smaller than the one caused by over- or underestimating energies (MacroState and MicroState versus NoDangle or OverDangle)*.

Models being so similar leads us to the question of runtime effort.

**Conclusion 3 ***Since results between MacroState and MicroState differ only marginally, MicroState may be used for probability calculation. The higher computational effort of MacroState is not justified*.

In the light of the previous conclusions we find:

**Conclusion 4 ***On longer sequences, the only remaining virtue of MacroState appears to be its ability to enumerate suboptimal structures with correct energies, and without redundancy*.

This answers the questions raised at the outset of this study.

### Evaluation of further models

Our evaluation has concentrated on the models underlying the programs RNAFOLD, RNASHAPES, and RNASUBOPT. There are many other folding programs out there. If these implementations adhere to the abstract models we present here in the form of tree grammars, our evaluation pertains to them as well. More likely, each implementation has its own peculiarities. In fact, one may think of extending our evaluation to models that are not based on thermodynamics at all, but are derived via machine learning techniques [25,26]. These programs could be evaluated in the setting of this study in one of two ways. Either, the program source code is extended by the computation of abstract shapes and their shape probabilities (a useful feature anyway), and applied to our data sets directly. Or, the model behind the program is extracted as a tree grammar, coded in Bellman's GAP, and combined with existing modules for shape abstraction and partition function computations. Depending on the model differences, extracting the grammar behind the code may come down to a few minor changes to the four models provided here.

Generally, the four models MacroState, MicroState, Overdangle and NoDangle are available as a starting point for future research into on thermodynamic RNA folding. Implemented in the Bellman's GAP language, these programs are especially easy to modify or extend, while the Bellman's GAP compiler provides automatic translation into efficient and correct dynamic programming algorithms. The complete source code of our four models is included in additional file 3.

### A new strategy for level-2 shape probabilities?

Our observations about the performance of shape level 2 gives rise to the investigation of a new strategy. Recall that level 2 gives much stronger information than levels 5 or even 3. Level 2 records not only the overall arrangement of helices, but also reports and distinguishes internal loops, 5' and 3' bulges.

Over all our data sets, consideration of (only) the five most likely level-2 shapes (using MicroState) reports the gold shape in 75% of the cases, while 25 level-2 shapes reach 90% coverage. However, the cost of level-2 shape analysis becomes prohibitive for longer sequences. Our data show a slowdown factor of 55 (for MicroState) over level-5 analysis, which should become even worse for longer sequences. Therefore, we conclude

**Conclusion 5 ***A strategy to efficiently compute level-2 shapes for long sequences is desirable*

Let us sketch a strategy how this can be achieved, borrowing ideas from the RAPIDSHAPES method [27]. Directly accessing the complete level-2 shape space of a long sequence appears infeasible. But we can compute a level-5 analysis at 90% or 100% coverage quickly, by reporting a small number top-ranking level-5 shapes (12 would suffice for 100% coverage on our data sets). For these shapes, we can generate a thermodynamic matcher [27] to perform a separate level-2 analysis within each of the reported level-5 shape classes. Generating such a matcher as a tree grammar, encoded in Bellman's GAP, plus its subsequent compilation has negligible runtime. This should reduce the computational effort (which results from the number of shapes) considerably. While this is not mathematically guaranteed to yield the most likely level-2 shape, the idea appears promising.

## Authors' contributions

SJ suggested to tackle the problem of model discrepancies empirically. SJ, RG and GS designed the study. CS prepared the data sets derived from 3D structural data, and GS provided background on the thermodynamic model. SJ implemented the four models and ran the evaluations. All authors closely cooperated in interpreting the results and writing the manuscript. All authors read and approved the final manuscript.

## Notes

^1^Our observations may pertain also to other popular programs such as MFOLD[28], UNAFOLD[29] and RNASTRUCTURE[30], but their folding space implementations have not been re-modeled here.

^2^One may view our re-engineering as adding shape probability functionality to the Vienna RNA package from outside.

^3^Similarly, stacking of helices [1,31,32] can further contribute free energy. This aspect is not considered here.

^4^RNAFOLD-manual: "-d or -d0 ignores dangling ends altogether (mostly for debugging)."

^5^RNAFOLD-manual: "With -d2 this check is ignored, dangling energies will be added for the bases adjacent to a helix on both sides in any case; this is the default for partition function folding (-p)."

^6^RNAFOLD-manual: "With -d1 only unpaired bases can participate in at most one dangling end, this is the default for mfe folding but unsupported for the partition function folding."

^7^A larger threshold will always help. However, one cannot tell whether this situation has occurred.

^8^Whether or not it is adequate in partition function computations to split a secondary structure into several microstates is an unresolved dispute among experts (M. Zuker, personal communication).

^9^This can be evaluated by experimental techniques [33], but sufficient data are not yet available.

^10^While in press, *Turner'2004 *energy parameters became available. Results for all evaluations are listed in additional file 4.

^11^It is not obvious how to convert our absolute distances into error rates. Remember that a mispredicted base pair can contribute a distance of 2 (cf. Figure 6). Assuming that predictions hold about the same number of base pairs as the gold structures (1593), the interval of possible distance scores is [0, 3186], from which the above percentages are derived.

^12^We also looked at four further UNAFOLD variants in dangle and no-dangle mode. Their behavior deviates considerably, which is explained by differences in the implemented energy model (M. Zuker, personal communication).

^13^In theory, these tables should be symmetric. We see a small asymmetry on the last decimal position in eight cases. This results from the fact that our programs - for better speed - ignore shapes with an initial probability less that 10^-6^. This means our resulting shape lists are nor perfectly identical in the low probability tail, and together with rounding errors, this leads to discrepancies ≤ 0.002.

## Supplementary Material

Additional file 1 **Measurements on Data Sets FR3D:3A, FR3D:4A and RNAstrand:91**. File "supplement.pdf" contains detailed results for the three mentioned data sets FR3D:3A, FR3D:4A and RNAstrand:91, which have not been shown in the main paper. We also provide four Venn diagrams to demonstrate overlaps between the data sets. Click here for file

Additional file 2 **Data Sets**. Archive "datasets.tgz" contains all four data sets DARTS, FR3D:3A, FR3D:4A and RNAstrand:91 as FASTA like files. Format description is given in additional file 1: "supplement.pdf". Click here for file

Additional file 3 **Source Code of all models**. The archive "fold-grammars.tgz" hold source code for all four models (NoDangle, OverDangle, MicroState and MacroState) in the Advanced Dynamic Programming language Bellman's GAP. Please see the enclosed readme file for further instructions on how to compile binaries. Click here for file

Additional file 4 **Evaluation results for Turner 2004 energy parameters**. File "turner2004.pdf" contains results for all our evaluations, but computed with the more recent *Turner 2004 *energy parameter set, which became available while our manuscript was in press. Click here for file

## References

[B1] Mathews D Sabina J Zuker M Turner D Expanded sequence dependence of thermodynamic parameters improves prediction of RNA secondary structure J Mol Biol 1999 288 911 940 10.1006/jmbi.1999.2700 10329189

[B2] Hofacker IL Fontana W Stadler PF Bonhoeffer SL Tacker M Schuster P Fast Folding and Comparison of RNA Secondary Structures Monatsh Chem 1994 125 167 188 10.1007/BF00818163

[B3] Giegerich R Voß B Rehmsmeier M Abstract shapes of RNA Nucleic Acids Research 2004 32 16 4843 10.1093/nar/gkh779 15371549 PMC519098

[B4] Wuchty S Fontana W Hofacker IL Schuster P Complete suboptimal folding of RNA and the stability of secondary structures Biopolymers 1999 49 2 145 165 10.1002/(SICI)1097-0282(199902)49:2<145::AID-BIP4>3.0.CO;2-G 10070264

[B5] Dowell R Eddy S Evaluation of several lightweight stochastic context-free grammars for RNA secondary structure prediction BMC Bioinformatics 2004 5 71 http://www.biomedcentral.com/1471-2105/5/71 10.1186/1471-2105-5-71 15180907 PMC442121

[B6] Voß B Giegerich R Rehmsmeier M Complete probabilistic analysis of RNA shapes BMC Biology 2006 4 5 10.1186/1741-7007-4-5 16480488 PMC1479382

[B7] Waterman M Introduction to computational biology. Maps, sequences and genomes 1995 London: Chapman & Hall

[B8] Nebel M Scheid A On quantitative effects of RNA shape abstraction Theory in Biosciences 2009 128 211 225 [10.1007/s12064-009-0074-z] 10.1007/s12064-009-0074-z 19756808

[B9] Janssen S Reeder J Giegerich R Shape based indexing for faster search of RNA family databases BMC Bioinformatics 2008 9 131+ 10.1186/1471-2105-9-131 18312625 PMC2277397

[B10] Borer P Dengler B Tinoco I Jr Uhlenbeck O Stability of ribonucleic acid double-stranded helices J Mol Biol 1974 86 843 853 10.1016/0022-2836(74)90357-X 4427357

[B11] Burkard M Kierzek R Turner D Thermodynamics of unpaired terminal nucleotides on short RNA helixes correlates with stacking at helix termini in larger RNAs J Mol Biol 1999 290 967 982 10.1006/jmbi.1999.2906 10438596

[B12] Ohmichi T Nakano S Miyoshi D Sugimoto N Long RNA dangling end has large energetic contribution to duplex stability J Am Chem Soc 2002 124 10367 10372 10.1021/ja0255406 12197739

[B13] Liu J Zhao L Xia T The dynamic structural basis of differential enhancement of conformational stability by 5'- and 3'-dangling ends in RNA Biochemistry 2008 47 5962 5975 10.1021/bi800210t 18457418

[B14] Giegerich R Meyer C Steffen P A discipline of dynamic programming over sequence data Science of Computer Programming 2004 51 3 215 263 10.1016/j.scico.2003.12.005

[B15] Giegerich R Sautho G Yield grammar analysis in the Bellman's GAP compiler Proceedings of the Eleventh Workshop on Language Descriptions, Tools and Applications 2011 LDTA 2011, ACM

[B16] Sauthoff G Janssen S Giegerich R Bellman's GAP - A Declarative Language for Dynamic Programming13th International ACM SIGPLAN Symposium on Principles and Practice of Declarative Programming, PPDP 2011 ACM 2011

[B17] Abraham M Dror O Nussinov R Wolfson H Analysis and classification of RNA tertiary structures RNA 2008 14 11 2274 10.1261/rna.853208 18824509 PMC2578864

[B18] Berman H Westbrook J Feng Z Gilliland G Bhat T Weissig H Shindyalov I Bourne P The protein data bank Nucleic Acids Res 2000 28 235 242 10.1093/nar/28.1.235 10592235 PMC102472

[B19] Rose P Beran B Bi C Bluhm W Dimitropoulos D Goodsell D Prlic A Quesada M Quinn G Westbrook J Young J Yukich B Zardecki C Berman H Bourne P The RCSB Protein Data Bank: redesigned web site and web services Nucleic Acids Res 2010 39 D392 401 21036868 10.1093/nar/gkq1021 PMC3013649

[B20] Sarver M Zirbel CL Stombaugh J Mokdad A Leontis NB FR3D: finding local and composite recurrent structural motifs in RNA 3D structures J Math Biol 2008 56 1-2 215 252 17694311 10.1007/s00285-007-0110-x PMC2837920

[B21] Stombaugh J Zirbel CL Westhof E Leontis NB Frequency and isostericity of RNA base pairs Nucleic Acids Res 2009 37 7 2294 2312 10.1093/nar/gkp011 19240142 PMC2673412

[B22] Gendron P Lemieux S Major F Quantitative analysis of nucleic acid three-dimensional structures J Mol Biol 2001 308 5 919 936 10.1006/jmbi.2001.4626 11352582

[B23] Andronescu M Bereg V Hoos H Condon A RNA STRAND: The RNA Secondary Structure and Statistical Analysis Database BMC Bioinformatics 2008 9 340 10.1186/1471-2105-9-340 18700982 PMC2536673

[B24] Hamada M Kiryu H Sato K Mituyama T Asai K Prediction of RNA secondary structure using generalized centroid estimators Bioinformatics 2009 25 4 465 473 10.1093/bioinformatics/btn601 19095700

[B25] Do CB Woods DA Batzoglou S CONTRAfold: RNA secondary structure prediction without physics-based models Bioinformatics 2006 22 14 e90 98 10.1093/bioinformatics/btl246 16873527

[B26] Andronescu M Condon A Hoos H Mathews DH Murphy KP Computational approaches for RNA energy parameter estimation RNA 2010 16 12 2304 2318 10.1261/rna.1950510 20940338 PMC2995392

[B27] Janssen S Giegerich R Faster computation of exact RNA shape probabilities Bioinformatics 2010 26 5 632 639 10.1093/bioinformatics/btq014 20080511 PMC2828121

[B28] Zuker M Mfold web server for nucleic acid folding and hybridization prediction Nucleic Acids Res 2003 31 3406 3415 10.1093/nar/gkg595 12824337 PMC169194

[B29] Markham NR Zuker M UNAFold: software for nucleic acid folding and hybridization Methods in molecular biology (Clifton, N.J.) 2008 453 3 31 10.1007/978-1-60327-429-6_1 18712296

[B30] Reuter J Mathews D RNAstructure: software for RNA secondary structure prediction and analysis BMC Bioinformatics 2010 11 129 10.1186/1471-2105-11-129 20230624 PMC2984261

[B31] Walter A Turner D Kim J Lyttle M Müller P Mathews D Zuker M Coaxial stacking of helixes enhances binding of oligoribonucleotides and improves predictions of RNA folding Proc Nat Acad Sci USA 1994 91 9218 9222 10.1073/pnas.91.20.9218 7524072 PMC44783

[B32] Xia T SantaLucia J Burkard M Kierzek R Schroeder S Jiao X Cox C Turner D Thermodynamic parameters for an expanded nearest-neighbor model for formation of RNA duplexes with Watson-Crick base pairs Biochemistry 1998 37 14719 14735 10.1021/bi9809425 9778347

[B33] Gherghe C Shajani Z Wilkinson K Varani G Weeks K Strong correlation between SHAPE chemistry and the generalized NMR order parameter (S2) in RNA J Am Chem Soc 2008 130 37 12244 5 10.1021/ja804541s 18710236 PMC2712629

[B34] Gardner P Giegerich R A comprehensive comparison of comparative RNA structure prediction approaches BMC Bioinformatics 2004 5 140 10.1186/1471-2105-5-140 15458580 PMC526219

