# Supplementary material for: Lost in folding space? Comparing four variants of the thermodynamic model for RNA secondary structure prediction
Source: BMC Bioinformatics. 2011 Nov 3;12:429. doi: 10.1186/1471-2105-12-429 (PMC3293930; doi:10.1186/1471-2105-12-429)
Supplement: Additional file 2 — Data Sets. Archive "datasets.tgz" contains all four data sets DARTS, FR3D:3A, FR3D:4A and RNAstrand:91 as FASTA like files. Format description is given in additional file 1: "supplement.pdf". [file 1471-2105-12-429-S2.PDF]

1 Supplemental material: results for test sets FR3D:3A, FR3D:4A and RNAstrand:91

This supplemental PDF reports results for those three test sets mentioned in the main paper but could not shown there in detail. Additionaly, you can find the source code of our implementations for NoDangle, OverDangle, MicroState and MacroState in the supplemental archive "fold-grammars.tgz".

1.1 Introduction

Creation of the data sets is explained in the 2011 BMC Bioinformatics paper "Lost in Folding Space? Comparing four variants of the thermodynamic model for RNA secondary structure prediction" by S. Janssen et al.. The format of the data is fasta-like:

- each data point consists of a sequence (one row, upper case letters `A`, `C`, `G` or `U`),
- headed by an ID which always starts with the fasta typical "`>`" character
- and two succeeding secondary structures. The first one is the MC-annotate interpretation of the PDB information (or in the "RNAstrand:91" case simply the data base structure)
- the second one is the "gold" structure, i.e. the PDB structure but without pseudoknots, isolated basepairs or too small hairpin loops. See paper chapter "Data Sets" for more details.

The RNAstrand:91 data set is a subset of the RNAstrand data base. We used the comfortable RNAstrand filtering interface with following options:

- Type = Any type. Since we don't care about the biological function of the non coding RNA.
- Source = Any source. All we can get.
- Length ≤ 200. Memory consumption for shape probability calculations is very high, especially in level 1 for MacroState. The worst case sequence example from our RNAstrand:91 set uses about 30GB memory.
- Validates by NMR or X-Ray = yes. We only accept wet lab confirmed structures as truth.
- Number of molecules in complex ≤ 1. Exclude inter molecular interactions.
- Fragment = no. Just look at complete molecules and thus complete structures.
- Duplicates = Non-redundant sequences only
- Stem features: Stem length, per stem ≥ 2. To be compatible with our grammars, which disallow lonely base pairs.
- Hairpin loop features: Number of free bases in hairpin loops, per hairpin loop ≥ 3. To be compatible with our grammars, which force a hairpin loop to have at least three unpaired bases.
- Pseudoknots: Number of pseudoknots per molecule: ≤ 0. Non of our grammars can handle pseudoknots.
- Additionally, we excluded sequences that contain other characters besides `A`, `C`, `G`, `U` or `T`. There are no energy parameters for degenerated bases.

The supplementary PDF contains results for the same analyses as in the paper, but for the three data sets (FR3D:3A, FR3D:4A and RNAstrand:91) that are not discusses there. The raw data sets themselves are contained in the "datasets.tgz" archive.

1.2 Data set overlaps

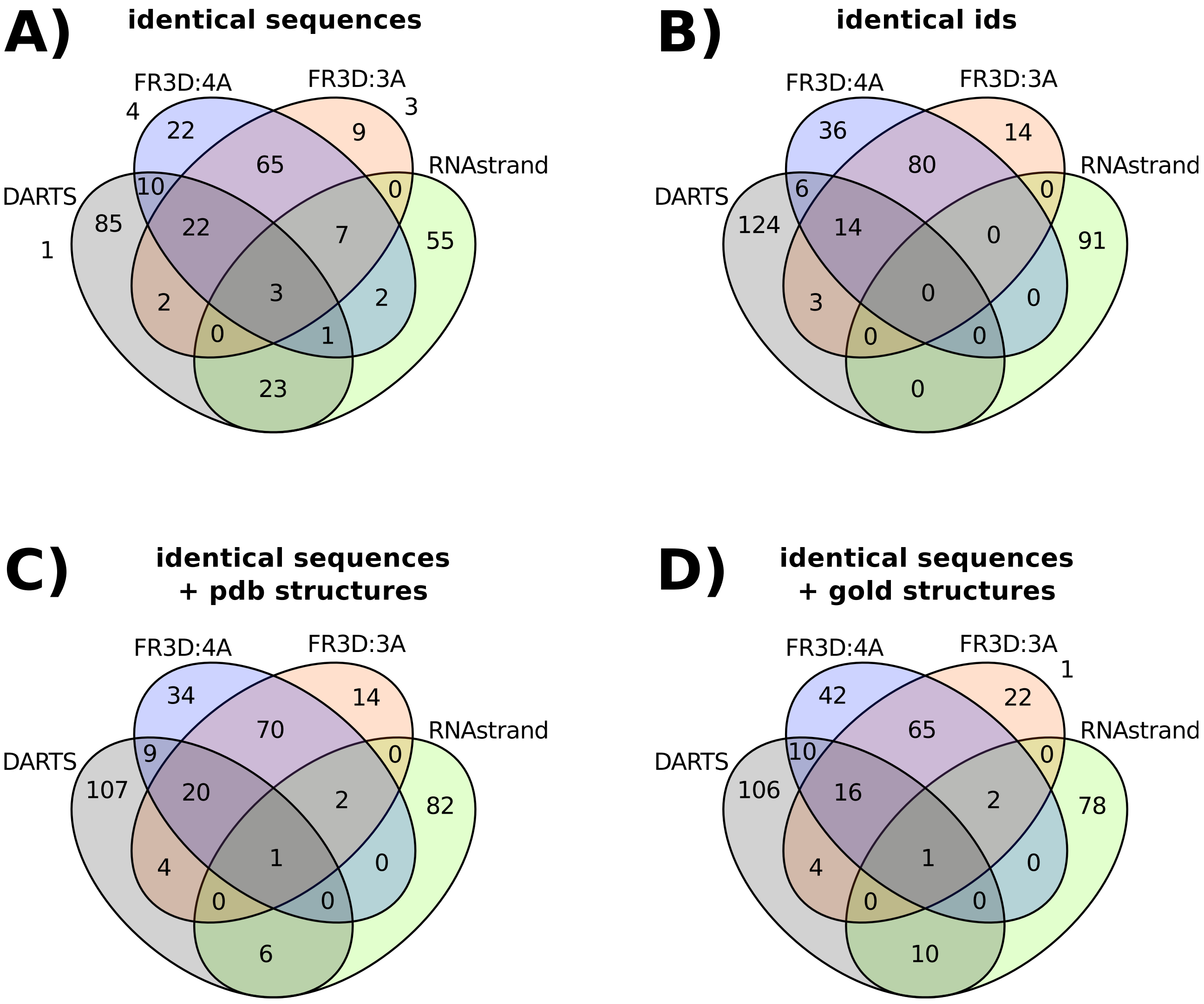

Figure 1: Overlaps of the four test sets DARTS, FR3D:3A, FR3D:4A and RNAstrand:91, shown as Venn diagrams. Since real RNA secondary structure data are rare and three of the four sets are contained in the PDB, we expect to see some overlaps between the different test sets. In Part A) we ask for identical primary sequences, independent of their annotated "PDB" or "gold" structure and independent of their header name. The later one is shown in Part B), where we ask for identical headers. Part C) accounts for identical primary sequences *and* identical "PDB" secondary structures, while Part D) looks at identical primary sequences *and* identical "gold" structures. Numbers outside of the elipses, as seen in Part A), indicate duplicates sequences in the respective data sets. As you can see in C) or D) these few cases of duplicate sequences do have different secondary structures.

1.3 FR3D:3A

Table 4 - Comparison of different MFE prediction programs.

Dataset: we use the 111 sequences from the FR3D:3A setTogether, all according “PDB” structures contain 1,483 base pairs. All “gold” structures have 1,410 base pairs.
Distance: One base pair set, i.e. secondary structure, is the reference (R: table columns), the other one is the prediction (P: table rows). Traditional base pair distance is defined as $|R \setminus P| + |P \setminus R|$. Following [29], we decide to allow additional base pairs in the prediction, as long as they are compatible with the reference, i.e. both bases are unpaired and the additional base pair does not introduce a pseudoknot in the reference. The set of compatible base pairs is $P^{-c} = P \setminus \{(a,b)|(a,b) \notin R \wedge (a,b) \text{ compatible to } R\}$. Then, our asymmetric base pair distance is: $|R \setminus P| + |P^{-c} \setminus R|$. Table values are the sums of base pair distances for all 111 sequences. In the case of co-optimal results, the one with the smallest distance to the reference is chosen.
Our distance function is rather strict and does not allow base pair slippage. If a gold base pair (i,j) is mispredicted as $(i+1,j)$, this contributes a distance of 2.
Programs: for each RNA sequence we called the programs with the following command line options: RNAfold (version 1.8.5): `echo sequence | RNAfold -noPS -noLP -dX`, where X is 0, 1 or 2. UNAFOLD (version 3.8): `hybrid-ss-min --suffix=DAT --mfold --NA=RNA --tmin=37 --tinc=1 --tmax=37 --sodium=1 --magnesium=0 --noisolate --nodangle tmpseqfile > /dev/null && ct2b.pl tmpseqfile.ct`, with and without the `--nodangle` switch, where “tmpseqfile” is a fasta file containing the sequence and “ct2b.pl” is a small Perl script from the Vienna Package, which converts RNA structures from “connect” to “dot-bracket” format. CentroidFold (version v0.0.9): `centroid_fold --engine=X tmpseqfile`, where X is the source of base pair probabilities and is either computed by RNAfold (McCaskill) or by CONTRAfold. Our ADP implementation of the four grammars “NoDangle”, “OverDangle”, “MicroState” and “MacroState” get the sequence as their sole input. The binaries can be built with the source code from the supplemental material and the Bellman’s GAP compiler.

reference (columns) / prediction (rows)

prediction	1:pd	2:go	3:RN	4:No	5:UN	6:RN	7:Ma	8:Mi	9:UN	10:RN	11:Ov	12:Ce	13:Ce
1: pdb structure	0	103											
2: gold structure	61	0											
3: RNAfold -d0	964	905	0	0	180	490	442	442	535	583	579	696	710
4: NoDangle	914	857	0	0	116	418	380	380	507	473	471	648	656
5: UNAfold –nodangle	954	895	174	110	0	550	477	477	422	567	563	695	724
6: RNAfold -d1	806	743	467	403	533	0	0	0	414	271	267	543	654
7: MacroState	752	689	425	363	465	0	0	0	348	209	205	491	592
8: MicroState	752	689	425	363	465	0	0	0	348	209	205	491	592
9: UNAfold	807	743	514	486	408	410	340	340	0	364	358	610	667
10: RNAfold -d2	729	668	593	491	581	299	233	233	396	0	0	514	619
11: OverDangle	723	662	589	489	577	295	229	229	390	0	0	508	611
12: CentroidFold McCaskill	291	239	502	452	506	359	315	315	457	302	302	0	211
13: CentroidFold CONTRAfold	421	365	508	452	527	487	421	421	510	420	416	219	0

Table 5 - Model similarity: shape probability shift

shape level 5

	Ma	Mi	Ov	No
Ma	0.000	0.072	0.081	0.166
Mi	0.072	0.000	0.076	0.228
Ov	0.081	0.076	0.000	0.216
No	0.166	0.228	0.213	0.000

shape level 4

	Ma	Mi	Ov	No
Ma	0.000	0.079	0.111	0.201
Mi	0.079	0.000	0.117	0.259
Ov	0.111	0.117	0.000	0.243
No	0.201	0.259	0.240	0.000

shape level 3

	Ma	Mi	Ov	No
Ma	0.000	0.085	0.125	0.215
Mi	0.085	0.000	0.139	0.276
Ov	0.125	0.139	0.000	0.253
No	0.215	0.276	0.249	0.000

shape level 2

	Ma	Mi	Ov	No
Ma	0.000	0.087	0.135	0.224
Mi	0.087	0.000	0.149	0.283
Ov	0.135	0.150	0.000	0.260
No	0.223	0.282	0.256	0.000

shape level 1

	Ma	Mi	Ov	No
Ma	0.000	0.128	0.190	0.304
Mi	0.134	0.000	0.236	0.382
Ov	0.191	0.235	0.000	0.304
No	0.303	0.376	0.299	0.000

Ma = **Ma**croStates
Mi = **Mi**croStates
Ov = **Ov**erDangle
No = **No**Dangle

Table 6 - Model similarity: average shape probability shift per shape

shape level 5

	Ma	Mi	Ov	No
Ma	0.000	0.021	0.020	0.051
Mi	0.019	0.000	0.020	0.068
Ov	0.017	0.019	0.000	0.056
No	0.058	0.081	0.074	0.000

shape level 4

	Ma	Mi	Ov	No
Ma	0.000	0.009	0.007	0.027
Mi	0.008	0.000	0.010	0.032
Ov	0.006	0.010	0.000	0.026
No	0.027	0.034	0.031	0.000

shape level 3

	Ma	Mi	Ov	No
Ma	0.000	0.008	0.005	0.025
Mi	0.007	0.000	0.010	0.029
Ov	0.005	0.009	0.000	0.024
No	0.025	0.031	0.028	0.000

shape level 2

	Ma	Mi	Ov	No
Ma	0.000	0.004	0.002	0.015
Mi	0.004	0.000	0.005	0.017
Ov	0.002	0.005	0.000	0.015
No	0.014	0.017	0.015	0.000

shape level 1

	Ma	Mi	Ov	No
Ma	0.000	0.005	0.001	0.011
Mi	0.006	0.000	0.006	0.017
Ov	0.001	0.006	0.000	0.012
No	0.012	0.016	0.012	0.000

Ma = **Ma**croStates
Mi = **Mi**croStates
Ov = **Ov**erDangle
No = **No**Dangle

Table 7 - Ratio of agreement between dominant shape and gold shape for the different grammars (columns) and different shape abstraction levels (rows).

Level	MacroState	MicroState	OverDangle	NoDangle
**5**	0.721	0.757	0.730	0.658
**4**	0.568	0.631	0.595	0.486
**3**	0.477	0.532	0.486	0.405
**2**	0.486	0.532	0.486	0.414
**1**	0.351	0.351	0.351	0.333

Table 8 - Positions of correct shapes.

Level	MacroState				MicroState				OverDangle				NoDangle			
	50%	75%	90%	100%	50%	75%	90%	100%	50%	75%	90%	100%	50%	75%	90%	100%
**5**	1	2	2	4	1	2	2	5	1	2	2	5	1	2	3	5
**4**	1	2	5	16	1	2	4	19	1	2	5	20	2	3	5	23
**3**	2	3	5	22	1	3	6	36	2	3	7	32	2	4	10	21
**2**	2	4	7	342	1	3	6	320	2	4	10	541	2	5	24	186
**1**	2	7	25	560	2	7	21	869	2	9	44	1730	3	16	65	640

Table 9 - Relative runtime. The MacroState level 5 value equals 17.03 seconds on an Intel® Xeon® CPU L5420 @ 2.50GHz.

Level	MacroState	MicroState	OverDangle	NoDangle
**5**	1.00	0.27	0.16	0.13
**4**	2.75	0.76	0.48	0.34
**3**	4.06	1.14	0.70	0.47
**2**	65.60	7.90	5.31	4.49
**1**	285.71	52.05	24.75	12.52

Table 10 - Relative memory. The MacroState level 5 value equals 29.1 MB resident set size.

Level	MacroState	MicroState	OverDangle	NoDangle
**5**	1.00	0.30	0.27	0.19
**4**	4.50	0.98	0.96	0.53
**3**	7.16	1.50	1.55	0.76
**2**	156.88	9.49	9.70	6.63
**1**	628.42	56.50	36.00	18.01

1.4 FR3D:4A

Table 4 - Comparison of different MFE prediction programs.

Dataset: we use the 136 sequences from the FR3D:4A setTogether, all according "PDB" structures contain 1,941 base pairs. All "gold" structures have 1,890 base pairs.
Distance: One base pair set, i.e. secondary structure, is the reference (R: table columns), the other one is the prediction (P: table rows). Traditional base pair distance is defined as $|R \setminus P| + |P \setminus R|$. Following [29], we decide to allow additional base pairs in the prediction, as long as they are compatible with the reference, i.e. both bases are unpaired and the additional base pair does not introduce a pseudoknot in the reference. The set of compatible base pairs is $P^{-c} = P \setminus \{(a,b)|(a,b) \notin R \wedge (a,b) \text{ compatible to } R\}$. Then, our asymmetric base pair distance is: $|R \setminus P| + |P^{-c} \setminus R|$. Table values are the sums of base pair distances for all 136 sequences. In the case of co-optimal results, the one with the smallest distance to the reference is chosen.
Our distance function is rather strict and does not allow base pair slippage. If a gold base pair (i, j) is mispredicted as $(i + 1, j)$, this contributes a distance of 2.
Programs: for each RNA sequence we called the programs with the following command line options: RNAFOLD (version 1.8.5): `echo sequence | RNAfold -noPS -noLP -dX`, where X is 0, 1 or 2. UNAFOLD (version 3.8): `hybrid-ss-min --suffix=DAT --mfold --NA=RNA --tmin=37 --tinc=1 --tmax=37 --sodium=1 --magnesium=0 --noisolate --nodangle tmpseqfile > /dev/null && ct2b.pl tmpseqfile.ct`, with and without the `--nodangle` switch, where "tmpseqfile" is a fasta file containing the sequence and "ct2b.pl" is a small Perl script from the Vienna Package, which converts RNA structures from "connect" to "dot-bracket" format. CENTROIDFOLD (version v0.0.9): `centroid_fold --engine=X tmpseqfile`, where X is the source of base pair probabilities and is either computed by RNAFOLD (McCaskill) or by CONTRAFOLD. Our ADP implementation of the four grammars "NoDangle", "OverDangle", "MicroState" and "MacroState" get the sequence as their sole input. The binaries can be built with the source code from the supplemental material and the Bellman's GAP compiler.

prediction \ reference	1:pd	2:go	3:RN	4:No	5:UN	6:RN	7:Ma	8:Mi	9:UN	10:RN	11:Ov	12:Ce	13:Ce
1: pdb structure	0	93											
2: gold structure	83	0											
3: RNAfold -d0	1332	1269	0	0	243	658	609	609	740	814	810	930	1001
4: NoDangle	1259	1188	0	0	167	572	533	533	698	680	678	859	925
5: UNAfold –nodangle	1312	1245	226	150	0	724	650	650	592	798	794	916	1006
6: RNAfold -d1	1088	1021	627	549	710	0	0	0	461	392	388	739	844
7: MacroState	1031	964	583	507	640	0	0	0	395	328	324	686	778
8: MicroState	1031	964	583	507	640	0	0	0	395	328	324	686	778
9: UNAfold	1135	1071	704	662	572	448	378	378	0	500	494	824	879
10: RNAfold -d2	1044	973	828	702	823	432	363	363	555	0	0	737	841
11: OverDangle	1028	955	824	700	819	428	359	359	549	0	0	719	818
12: CentroidFold McCaskill	430	363	674	600	675	500	456	456	614	445	430	0	272
13: CentroidFold CONTRAfold	611	539	774	694	792	655	587	587	707	595	574	310	0

Table 5 - Model similarity: shape probability shift

	Ma	Mi	Ov	No
Ma	0.000	0.066	0.094	0.175
Mi	0.066	0.000	0.081	0.235
Ov	0.094	0.081	0.000	0.237
No	0.175	0.234	0.236	0.000

shape level 5

	Ma	Mi	Ov	No
Ma	0.000	0.073	0.122	0.204
Mi	0.073	0.000	0.122	0.264
Ov	0.122	0.122	0.000	0.259
No	0.204	0.264	0.257	0.000

shape level 4

	Ma	Mi	Ov	No
Ma	0.000	0.080	0.144	0.223
Mi	0.080	0.000	0.152	0.285
Ov	0.145	0.152	0.000	0.269
No	0.223	0.285	0.267	0.000

shape level 3

	Ma	Mi	Ov	No
Ma	0.000	0.082	0.154	0.232
Mi	0.082	0.000	0.161	0.292
Ov	0.154	0.162	0.000	0.276
No	0.231	0.292	0.274	0.000

shape level 2

	Ma	Mi	Ov	No
Ma	0.000	0.125	0.226	0.317
Mi	0.130	0.000	0.268	0.398
Ov	0.226	0.267	0.000	0.316
No	0.317	0.394	0.313	0.000

shape level 1

Ma = **Ma**croStates
Mi = **Mi**croStates
Ov = **Ov**erDangle
No = **No**Dangle

Table 6 - Model similarity: average shape probability shift per shape

	Ma	Mi	Ov	No
Ma	0.000	0.019	0.024	0.053
Mi	0.018	0.000	0.020	0.067
Ov	0.018	0.019	0.000	0.058
No	0.059	0.080	0.077	0.000

shape level 5

	Ma	Mi	Ov	No
Ma	0.000	0.007	0.008	0.022
Mi	0.007	0.000	0.009	0.027
Ov	0.006	0.009	0.000	0.021
No	0.022	0.029	0.026	0.000

shape level 4

	Ma	Mi	Ov	No
Ma	0.000	0.006	0.006	0.018
Mi	0.006	0.000	0.009	0.021
Ov	0.005	0.008	0.000	0.017
No	0.018	0.023	0.021	0.000

shape level 3

	Ma	Mi	Ov	No
Ma	0.000	0.003	0.002	0.008
Mi	0.003	0.000	0.004	0.011
Ov	0.002	0.004	0.000	0.009
No	0.008	0.010	0.008	0.000

shape level 2

	Ma	Mi	Ov	No
Ma	0.000	0.004	0.001	0.006
Mi	0.004	0.000	0.005	0.011
Ov	0.001	0.005	0.000	0.007
No	0.007	0.011	0.007	0.000

shape level 1

Ma = **Ma**croStates
Mi = **Mi**croStates
Ov = **Ov**erDangle
No = **No**Dangle

Table 7 - Ratio of agreement between dominant shape and gold shape for the different grammars (columns) and different shape abstraction levels (rows).

Level	MacroState	MicroState	OverDangle	NoDangle
**5**	0.711	0.756	0.711	0.615
**4**	0.533	0.570	0.533	0.430
**3**	0.489	0.519	0.459	0.385
**2**	0.489	0.496	0.452	0.393
**1**	0.348	0.319	0.341	0.311

Table 8 - Positions of correct shapes.

Level	MacroState				MicroState				OverDangle				NoDangle			
	50%	75%	90%	100%	50%	75%	90%	100%	50%	75%	90%	100%	50%	75%	90%	100%
**5**	1	2	2	7	1	2	3	6	1	2	3	6	2	2	3	7
**4**	1	3	6	30	1	3	5	28	1	3	6	39	2	4	10	36
**3**	2	3	8	41	1	3	6	46	2	4	8	41	2	5	14	38
**2**	2	5	16	217	2	4	13	204	2	4	15	9939	2	8	49	288
**1**	3	11	54	18126	3	11	68	5256	3	11	100	14811	3	28	175	3445

Table 9 - Relative runtime. The MacroState level 5 value equals 25.86 seconds on an Intel® Xeon® CPU L5420 @ 2.50GHz.

Level	MacroState	MicroState	OverDangle	NoDangle
**5**	1.00	0.26	0.16	0.13
**4**	2.93	0.77	0.48	0.34
**3**	4.23	1.16	0.68	0.47
**2**	86.77	9.51	5.84	4.96
**1**	351.46	64.90	26.42	15.29

Table 10 - Relative memory. The MacroState level 5 value equals 30.7 MB resident set size.

Level	MacroState	MicroState	OverDangle	NoDangle
**5**	1.00	0.26	0.27	0.21
**4**	4.11	0.89	0.82	0.50
**3**	6.12	1.14	1.16	0.64
**2**	168.89	7.12	8.41	6.09
**1**	794.08	51.26	47.97	17.82

1.5 RNAstrand:91

Table 4 - Comparison of different MFE prediction programs.

Dataset: we use the 91 sequences from the rnastrand setTogether, all according "PDB" structures contain 1,247 base pairs. All "gold" structures have 1,247 base pairs.
Distance: One base pair set, i.e. secondary structure, is the reference (R: table columns), the other one is the prediction (P: table rows). Traditional base pair distance is defined as $|R \setminus P| + |P \setminus R|$. Following [29], we decide to allow additional base pairs in the prediction, as long as they are compatible with the reference, i.e. both bases are unpaired and the additional base pair does not introduce a pseudoknot in the reference. The set of compatible base pairs is $P^{-c} = P \setminus \{(a,b)|(a,b) \notin R \wedge (a,b) \text{ compatible to } R\}$. Then, our asymmetric base pair distance is: $|R \setminus P| + |P^{-c} \setminus R|$. Table values are the sums of base pair distances for all 91 sequences. In the case of co-optimal results, the one with the smallest distance to the reference is chosen.
Our distance function is rather strict and does not allow base pair slippage. If a gold base pair (i, j) is mispredicted as $(i + 1, j)$, this contributes a distance of 2.
Programs: for each RNA sequence we called the programs with the following command line options: RNAFOLD (version 1.8.5): `echo sequence | RNAfold -noPS -noLP -dX`, where X is 0, 1 or 2. UNAFOLD (version 3.8): `hybrid-ss-min --suffix=DAT --mfold --NA=RNA --tmin=37 --tinc=1 --tmax=37 --sodium=1 --magnesium=0 --noisolate --nodangle tmpseqfile > /dev/null && ct2b.pl tmpseqfile.ct`, with and without the `--nodangle` switch, where "tmpseqfile" is a fasta file containing the sequence and "ct2b.pl" is a small Perl script from the Vienna Package, which converts RNA structures from "connect" to "dot-bracket" format. CENTROIDFOLD (version v0.0.9): `centroid_fold --engine=X tmpseqfile`, where X is the source of base pair probabilities and is either computed by RNAFOLD (McCaskill) or by CONTRAFOLD. Our ADP implementation of the four grammars "NoDangle", "OverDangle", "MicroState" and "MacroState" get the sequence as their sole input. The binaries can be built with the source code from the supplemental material and the Bellman's GAP compiler.

prediction \ reference	1:pd	2:go	3:RN	4:No	5:UN	6:RN	7:Ma	8:Mi	9:UN	10:RN	11:Ov	12:Ce	13:Ce
1: pdb structure	0	0											
2: gold structure	0	0											
3: RNAfold -d0	562	562	0	0	104	292	239	239	243	231	230	367	354
4: NoDangle	547	547	0	0	100	270	222	222	239	209	209	356	338
5: UNAfold –nodangle	542	542	95	91	0	305	248	248	148	236	231	348	364
6: RNAfold -d1	536	536	287	269	310	0	0	0	202	119	119	344	419
7: MacroState	481	481	234	217	253	0	0	0	145	79	79	293	365
8: MicroState	481	481	234	217	253	0	0	0	145	79	79	293	365
9: UNAfold	543	543	231	227	144	194	137	137	0	190	185	348	362
10: RNAfold -d2	478	478	228	210	243	122	82	82	201	0	0	287	346
11: OverDangle	475	475	227	210	238	122	82	82	196	0	0	284	340
12: CentroidFold McCaskill	196	196	257	243	245	220	174	174	241	155	153	0	106
13: CentroidFold CONTRAfold	241	241	260	243	278	328	279	279	275	252	248	132	0

Table 5 - Model similarity: shape probability shift

	Ma	Mi	Ov	No
Ma	0.000	0.025	0.048	0.090
Mi	0.025	0.000	0.039	0.113
Ov	0.048	0.039	0.000	0.117
No	0.090	0.114	0.117	0.000

shape level 5

	Ma	Mi	Ov	No
Ma	0.000	0.030	0.054	0.101
Mi	0.030	0.000	0.049	0.126
Ov	0.054	0.049	0.000	0.126
No	0.101	0.127	0.126	0.000

shape level 4

	Ma	Mi	Ov	No
Ma	0.000	0.032	0.064	0.105
Mi	0.032	0.000	0.061	0.130
Ov	0.064	0.061	0.000	0.131
No	0.105	0.130	0.131	0.000

shape level 3

	Ma	Mi	Ov	No
Ma	0.000	0.036	0.065	0.112
Mi	0.036	0.000	0.065	0.139
Ov	0.065	0.065	0.000	0.135
No	0.112	0.140	0.136	0.000

shape level 2

	Ma	Mi	Ov	No
Ma	0.000	0.068	0.097	0.169
Mi	0.075	0.000	0.125	0.216
Ov	0.097	0.122	0.000	0.169
No	0.169	0.213	0.168	0.000

shape level 1

Ma = **Ma**croStates
Mi = **Mi**croStates
Ov = **Ov**erDangle
No = **No**Dangle

Table 6 - Model similarity: average shape probability shift per shape

	Ma	Mi	Ov	No
Ma	0.000	0.007	0.011	0.027
Mi	0.006	0.000	0.008	0.031
Ov	0.009	0.007	0.000	0.028
No	0.027	0.035	0.036	0.000

shape level 5

	Ma	Mi	Ov	No
Ma	0.000	0.003	0.003	0.012
Mi	0.003	0.000	0.004	0.014
Ov	0.002	0.003	0.000	0.011
No	0.011	0.014	0.013	0.000

shape level 4

	Ma	Mi	Ov	No
Ma	0.000	0.003	0.003	0.012
Mi	0.003	0.000	0.004	0.014
Ov	0.002	0.004	0.000	0.011
No	0.011	0.015	0.013	0.000

shape level 3

	Ma	Mi	Ov	No
Ma	0.000	0.001	0.001	0.006
Mi	0.001	0.000	0.002	0.007
Ov	0.001	0.002	0.000	0.006
No	0.006	0.007	0.006	0.000

shape level 2

	Ma	Mi	Ov	No
Ma	0.000	0.004	0.000	0.006
Mi	0.005	0.000	0.005	0.011
Ov	0.000	0.004	0.000	0.007
No	0.007	0.011	0.007	0.000

shape level 1

Ma = **Ma**croStates
Mi = **Mi**croStates
Ov = **Ov**erDangle
No = **No**Dangle

Table 7 - Ratio of agreement between dominant shape and gold shape for the different grammars (columns) and different shape abstraction levels (rows).

Level	MacroState	MicroState	OverDangle	NoDangle
**5**	0.888	0.910	0.899	0.854
**4**	0.629	0.629	0.607	0.596
**3**	0.685	0.685	0.663	0.652
**2**	0.584	0.584	0.562	0.562
**1**	0.517	0.517	0.494	0.483

Table 8 - Positions of correct shapes.

Level	MacroState				MicroState				OverDangle				NoDangle			
	50%	75%	90%	100%	50%	75%	90%	100%	50%	75%	90%	100%	50%	75%	90%	100%
**5**	1	2	2	8	1	2	2	12	1	2	2	10	2	2	2	4
**4**	1	2	5	86	1	2	6	124	1	2	5	217	1	3	8	92
**3**	1	2	4	261	1	2	3	192	1	2	4	505	1	2	5	114
**2**	1	3	12	590	1	2	12	355	1	3	13	1075	1	5	58	891
**1**	1	28	534	2861	1	20	290	1586	2	22	375	4336	2	22	370	1764

Table 9 - Relative runtime. The MacroState level 5 value equals 10.67 seconds on an Intel® Xeon® CPU L5420 @ 2.50GHz.

Level	MacroState	MicroState	OverDangle	NoDangle
**5**	1.00	0.27	0.16	0.13
**4**	3.33	0.89	0.54	0.41
**3**	4.98	1.39	0.82	0.60
**2**	128.57	12.34	7.70	8.16
**1**	612.08	116.38	50.68	31.65

Table 10 - Relative memory. The MacroState level 5 value equals 32.7 MB resident set size.

Level	MacroState	MicroState	OverDangle	NoDangle
**5**	1.00	0.26	0.26	0.22
**4**	4.07	0.69	0.78	0.59
**3**	6.01	1.01	1.19	0.82
**2**	146.55	6.69	8.19	8.53
**1**	758.91	47.55	47.44	26.78

2 Alternative measures to SPS

One of our peer reviewers had the following concern about "Evaluating models for partition function"

> As an evaluation measure to evaluate this, the authors employed the shape probability shift (SPS). I am not really sure that this measure is the best measure to evaluate estimated probability distribution. (Of course, I think that SPS is one of the measures to evaluate base paring probability matrix or distribution of secondary structures).
> How about using the squared difference between two base paring probability matrices?
>
> OR
>
> When you would like to compare two probability distributions of secondary structures, how about using the Kullback-Leibler divergence, which is a distance between two probability distributions? I think that, by using this, you can directly compare the model similarity in terms of the similarity of probability distributions. (I agree that the evaluation of base paring probability matrix or distribution of secondary structures is quite difficult, because nobody can know the correct distributions of possible secondary structures.)
> Anyway, the authors should clarify that SPS is better measure than the above measures.

To adequately respond to this point, we investigated the correlation between "Shape Probability Shift (SPS)" and the suggested alternative measures "Squared Differences (SD)" and "Kullback-Leibler divergence (KL)". In the following we first give definitions for SD, KL and "Pearson product-moment correlation coefficient (r)". Subsequently, we report our results for all four testsets DARTS, FR3D:3A, FR3D:4A and RNAstrand:91.

2.1 Definitions

$SD_{A,B}(x)$ is the Squared Difference between models A and B for sequence x. We use the following definition:

$$SD_{A,B}(x) = \sum_{0 \leq i < j < |x|} \left(BPprob_A(x_{i,j}) - BPprob_B(x_{i,j})\right)^2 \tag{1}$$

where $BPprob_A(x_{i,j})$ is the probability of the presence of a base pair between nucleotides i and j of sequence x (as long as we don't have a generic implementation for the outside algorithm in Bellman's GAP, we cannot compute base pair probabilities. Thus for this evaluation we use the "dot.ps" files, generated by RNAFOLD via `echo "x" | RNAfold -p -dy`, where y is either 0 or 2. Since RNAFOLD cannot compute MicroState nor MacroState partition functions, we are left to NoDangle (y=0) and OverDangle (y=2)).
$KL_{A,B}(x)$ is the Kullback-Leibler divergence between models A and B for sequence x. Please note, that this measure is not symmetric. We use the following definition:

$$KL_{A,B}(x) = \sum_{0 \leq i < j < |x|} BPprob_A(x_{i,j}) \cdot log_2 \left(\frac{BPprob_A(x_{i,j})}{BPprob_B(x_{i,j})}\right) \tag{2}$$

In order to judge the different measures (SD, KL and SPS), averaging over all sequences of the testset D would not be very informative. Instead we apply the correlation coefficient, namely the "Pearson product-moment correlation coefficient" defined as follow:

$$r_{M,N} = \frac{\sum_{i=1}^{n} \left(M_i - \bar{M}\right) \cdot \left(N_i - \bar{N}\right)}{\sqrt{\sum_{i=1}^{n} \left(M_i - \bar{M}\right)^2} \cdot \sqrt{\sum_{i=1}^{n} \left(N_i - \bar{N}\right)^2}} \tag{3}$$

2.2 Results

2.2.1 DARTS

Table 1 - Alternative SPS measures.

Dataset: we use all 147 sequences from the DARTS set.
Content: we computed the correlation coefficient r for each pairwise combination of available measures. Since KL and SPS are not symmetric we include both directions for all measures, e.g. $KL_{A,B}$ and $KL_{B,A}$. The SPS depends on the selected shape level l, thus we have five different SPS_l. In total we have 14 measures.

N=NoDangle (columns); O=OverDangle (rows)

	1:SD	2:SD	3:KL	4:KL	5:SP	6:SP	7:SP	8:SP	9:SP	10:SP	11:SP	12:SP	13:SP	14:SP
1: SD$_{N,O}$	1.000	1.000	0.896	0.795	0.806	0.809	0.790	0.793	0.786	0.788	0.780	0.781	0.752	0.750
2: SD$_{O,N}$	1.000	1.000	0.896	0.795	0.806	0.809	0.790	0.793	0.786	0.788	0.780	0.781	0.752	0.750
3: KL$_{N,O}$	0.896	0.896	1.000	0.515	0.660	0.657	0.639	0.635	0.638	0.634	0.632	0.625	0.605	0.597
4: KL$_{O,N}$	0.795	0.795	0.515	1.000	0.764	0.776	0.745	0.758	0.743	0.756	0.736	0.750	0.706	0.718
5: SPS_5$_{N,O}$	0.806	0.806	0.660	0.764	1.000	0.999	0.961	0.961	0.956	0.956	0.945	0.945	0.894	0.896
6: SPS_5$_{O,N}$	0.809	0.809	0.657	0.776	0.999	1.000	0.961	0.962	0.956	0.957	0.944	0.945	0.893	0.896
7: SPS_4$_{N,O}$	0.790	0.790	0.639	0.745	0.961	0.961	1.000	0.999	0.990	0.989	0.990	0.989	0.952	0.953
8: SPS_4$_{O,N}$	0.793	0.793	0.635	0.758	0.961	0.962	0.999	1.000	0.989	0.990	0.989	0.989	0.951	0.953
9: SPS_3$_{N,O}$	0.786	0.786	0.638	0.743	0.956	0.956	0.990	0.989	1.000	0.999	0.997	0.996	0.959	0.960
10: SPS_3$_{O,N}$	0.788	0.788	0.634	0.756	0.956	0.957	0.989	0.990	0.999	1.000	0.996	0.997	0.958	0.960
11: SPS_2$_{N,O}$	0.780	0.780	0.632	0.736	0.945	0.944	0.990	0.989	0.997	0.996	1.000	0.999	0.965	0.965
12: SPS_2$_{O,N}$	0.781	0.781	0.625	0.750	0.945	0.945	0.989	0.989	0.996	0.997	0.999	1.000	0.964	0.965
13: SPS_1$_{N,O}$	0.752	0.752	0.605	0.706	0.894	0.893	0.952	0.951	0.959	0.958	0.965	0.964	1.000	0.999
14: SPS_1$_{O,N}$	0.750	0.750	0.597	0.718	0.896	0.896	0.953	0.953	0.960	0.960	0.965	0.965	0.999	1.000

2.2.2 FR3D:3A

Table 2 - Alternative SPS measures.

Dataset: we use all 111 sequences from the FR3D:3A set.
Content: we computed the correlation coefficient r for each pairwise combination of available measures. Since KL and SPS are not symmetric we include both directions for all measures, e.g. $KL_{A,B}$ and $KL_{B,A}$. The SPS depends on the selected shape level l, thus we have five different SPS_l. In total we have 14 measures.

N=NoDangle (columns); O=OverDangle (rows)

	1:SD	2:SD	3:KL	4:KL	5:SP	6:SP	7:SP	8:SP	9:SP	10:SP	11:SP	12:SP	13:SP	14:SP
1: $SD_{N,O}$	1.000	1.000	0.846	0.839	0.861	0.869	0.843	0.854	0.836	0.847	0.830	0.842	0.793	0.809
2: $SD_{O,N}$	1.000	1.000	0.846	0.839	0.861	0.869	0.843	0.854	0.836	0.847	0.830	0.842	0.793	0.809
3: $KL_{N,O}$	0.846	0.846	1.000	0.502	0.778	0.776	0.756	0.754	0.754	0.751	0.748	0.744	0.725	0.721
4: $KL_{O,N}$	0.839	0.839	0.502	1.000	0.715	0.730	0.708	0.727	0.695	0.716	0.696	0.720	0.652	0.686
5: $SPS\_5_{N,O}$	0.861	0.861	0.778	0.715	1.000	0.999	0.963	0.962	0.959	0.959	0.944	0.944	0.895	0.898
6: $SPS\_5_{O,N}$	0.869	0.869	0.776	0.730	0.999	1.000	0.961	0.963	0.957	0.960	0.942	0.945	0.892	0.898
7: $SPS\_4_{N,O}$	0.843	0.843	0.756	0.708	0.963	0.961	1.000	0.998	0.981	0.980	0.985	0.984	0.943	0.945
8: $SPS\_4_{O,N}$	0.854	0.854	0.754	0.727	0.962	0.963	0.998	1.000	0.980	0.982	0.984	0.986	0.941	0.946
9: $SPS\_3_{N,O}$	0.836	0.836	0.754	0.695	0.959	0.957	0.981	0.980	1.000	0.998	0.994	0.993	0.955	0.956
10: $SPS\_3_{O,N}$	0.847	0.847	0.751	0.716	0.959	0.960	0.980	0.982	0.998	1.000	0.992	0.994	0.952	0.957
11: $SPS\_2_{N,O}$	0.830	0.830	0.748	0.696	0.944	0.942	0.985	0.984	0.994	0.992	1.000	0.998	0.962	0.963
12: $SPS\_2_{O,N}$	0.842	0.842	0.744	0.720	0.944	0.945	0.984	0.986	0.993	0.994	0.998	1.000	0.959	0.964
13: $SPS\_1_{N,O}$	0.793	0.793	0.725	0.652	0.895	0.892	0.943	0.941	0.955	0.952	0.962	0.959	1.000	0.998
14: $SPS\_1_{O,N}$	0.809	0.809	0.721	0.686	0.898	0.898	0.945	0.946	0.956	0.957	0.963	0.964	0.998	1.000

2.2.3 FR3D:4A

Table 3 - Alternative SPS measures.

Dataset: we use all 136 sequences from the FR3D:4A set.
Content: we computed the correlation coefficient r for each pairwise combination of available measures. Since KL and SPS are not symmetric we include both directions for all measures, e.g. $KL_{A,B}$ and $KL_{B,A}$. The SPS depends on the selected shape level l, thus we have five different SPS_l. In total we have 14 measures.

N=NoDangle (columns); O=OverDangle (rows)

	1:SD	2:SD	3:KL	4:KL	5:SP	6:SP	7:SP	8:SP	9:SP	10:SP	11:SP	12:SP	13:SP	14:SP
1: $SD_{N,O}$	1.000	1.000	0.820	0.789	0.824	0.829	0.823	0.828	0.816	0.821	0.811	0.816	0.787	0.791
2: $SD_{O,N}$	1.000	1.000	0.820	0.789	0.824	0.829	0.823	0.828	0.816	0.821	0.811	0.816	0.787	0.791
3: $KL_{N,O}$	0.820	0.820	1.000	0.406	0.700	0.699	0.688	0.687	0.695	0.694	0.688	0.685	0.669	0.663
4: $KL_{O,N}$	0.789	0.789	0.406	1.000	0.678	0.685	0.687	0.694	0.668	0.677	0.672	0.682	0.642	0.655
5: $SPS\_5_{N,O}$	0.824	0.824	0.700	0.678	1.000	0.999	0.977	0.976	0.974	0.974	0.961	0.961	0.924	0.926
6: $SPS\_5_{O,N}$	0.829	0.829	0.699	0.685	0.999	1.000	0.976	0.977	0.973	0.974	0.960	0.961	0.923	0.926
7: $SPS\_4_{N,O}$	0.823	0.823	0.688	0.687	0.977	0.976	1.000	0.999	0.986	0.986	0.988	0.988	0.960	0.961
8: $SPS\_4_{O,N}$	0.828	0.828	0.687	0.694	0.976	0.977	0.999	1.000	0.986	0.986	0.987	0.988	0.959	0.961
9: $SPS\_3_{N,O}$	0.816	0.816	0.695	0.668	0.974	0.973	0.986	0.986	1.000	0.999	0.995	0.995	0.971	0.971
10: $SPS\_3_{O,N}$	0.821	0.821	0.694	0.677	0.974	0.974	0.986	0.986	0.999	1.000	0.995	0.995	0.969	0.972
11: $SPS\_2_{N,O}$	0.811	0.811	0.688	0.672	0.961	0.960	0.988	0.987	0.995	0.995	1.000	0.999	0.977	0.978
12: $SPS\_2_{O,N}$	0.816	0.816	0.685	0.682	0.961	0.961	0.988	0.988	0.995	0.995	0.999	1.000	0.976	0.978
13: $SPS\_1_{N,O}$	0.787	0.787	0.669	0.642	0.924	0.923	0.960	0.959	0.971	0.969	0.977	0.976	1.000	0.999
14: $SPS\_1_{O,N}$	0.791	0.791	0.663	0.655	0.926	0.926	0.961	0.961	0.971	0.972	0.978	0.978	0.999	1.000

2.2.4 RNAstrand:91

Table 4 - Alternative SPS measures.

Dataset: we use all 91 sequences from the rnastrand set.
Content: we computed the correlation coefficient r for each pairwise combination of available measures. Since KL and SPS are not symmetric we include both directions for all measures, e.g. $KL_{A,B}$ and $KL_{B,A}$. The SPS depends on the selected shape level l, thus we have five different SPS_l. In total we have 14 measures.

O=OverDangle	N=NoDangle													
	1:SD	2:SD	3:KL	4:KL	5:SP	6:SP	7:SP	8:SP	9:SP	10:SP	11:SP	12:SP	13:SP	14:SP
1: SD$_{N,O}$	1.000	1.000	0.782	0.845	0.882	0.884	0.876	0.879	0.868	0.872	0.866	0.869	0.798	0.804
2: SD$_{O,N}$	1.000	1.000	0.782	0.845	0.882	0.884	0.876	0.879	0.868	0.872	0.866	0.869	0.798	0.804
3: KL$_{N,O}$	0.782	0.782	1.000	0.445	0.832	0.832	0.832	0.831	0.838	0.837	0.838	0.836	0.790	0.785
4: KL$_{O,N}$	0.845	0.845	0.445	1.000	0.701	0.704	0.696	0.699	0.684	0.689	0.682	0.688	0.615	0.630
5: SPS_5$_{N,O}$	0.882	0.882	0.832	0.701	1.000	1.000	0.988	0.988	0.981	0.981	0.970	0.970	0.897	0.898
6: SPS_5$_{O,N}$	0.884	0.884	0.832	0.704	1.000	1.000	0.988	0.988	0.981	0.981	0.970	0.970	0.897	0.898
7: SPS_4$_{N,O}$	0.876	0.876	0.832	0.696	0.988	0.988	1.000	1.000	0.991	0.991	0.988	0.987	0.931	0.931
8: SPS_4$_{O,N}$	0.879	0.879	0.831	0.699	0.988	0.988	1.000	1.000	0.991	0.991	0.988	0.988	0.931	0.932
9: SPS_3$_{N,O}$	0.868	0.868	0.838	0.684	0.981	0.981	0.991	0.991	1.000	1.000	0.996	0.996	0.940	0.940
10: SPS_3$_{O,N}$	0.872	0.872	0.837	0.689	0.981	0.981	0.991	0.991	1.000	1.000	0.996	0.996	0.939	0.940
11: SPS_2$_{N,O}$	0.866	0.866	0.838	0.682	0.970	0.970	0.988	0.988	0.996	0.996	1.000	1.000	0.958	0.959
12: SPS_2$_{O,N}$	0.869	0.869	0.836	0.688	0.970	0.970	0.987	0.988	0.996	0.996	1.000	1.000	0.958	0.959
13: SPS_1$_{N,O}$	0.798	0.798	0.790	0.615	0.897	0.897	0.931	0.931	0.940	0.939	0.958	0.958	1.000	1.000
14: SPS_1$_{O,N}$	0.804	0.804	0.785	0.630	0.898	0.898	0.931	0.932	0.940	0.940	0.959	0.959	1.000	1.000

References

[29] Gardner, Paul P. and Giegerich, Robert. A comprehensive comparison of comparative RNA structure prediction approaches. *BMC Bioinformatics*, 5(1):140, 2004.
