# Supplementary material for: Lost in folding space? Comparing four variants of the thermodynamic model for RNA secondary structure prediction
Source: BMC Bioinformatics. 2011 Nov 3;12:429. doi: 10.1186/1471-2105-12-429 (PMC3293930; doi:10.1186/1471-2105-12-429)
Supplement: Additional file 4 — Evaluation results for Turner 2004 energy parameters. File "turner2004.pdf" contains results for all our evaluations, but computed with the more recent Turner 2004 energy parameter set, which became available while our manuscript was in press. [file 1471-2105-12-429-S4.PDF]

While our manuscript was in press, the new *Turner 2004* energy parameters became available. Thus, we repeated all evaluations with the same test-sets but updated energy parameters. Since we did not see surprisingly different results, compared to the *Turner1999* parameters used in the paper and due to the lack of time, we only list the results here as additional material.

1 DARTS set

Table 1 - Comparison of different MFE prediction programs.

Using the 152 sequences from the DARTS set, we repeated the same evaluation as shown the the Table 4 of the paper, but with *Turner 2004* energy parameters.

reference

prediction	1:pd	2:go	3:RN	4:no	5:UN	6:RN	7:ma	8:mi	9:UN	10:RN	11:ov
1: pdb structure	0	44									
2: gold structure	30	0									
3: RNAfold -d0	725	700	0	0	597	344	340	344	585	352	351
4: NoDangle	723	698	0	0	595	342	338	342	581	352	351
5: UNAfold -nodangle	801	772	618	612	0	587	582	573	612	541	527
6: RNAfold -d1	557	527	343	337	563	0	3	0	357	170	161
7: MacroState	555	525	340	334	561	1	0	1	353	168	159
8: MicroState	557	527	343	337	552	0	3	0	340	170	161
9: UNAfold	584	555	580	574	578	351	346	330	0	349	342
10: RNAfold -d2	474	443	362	358	529	181	181	181	365	0	0
11: OverDangle	472	441	362	358	521	172	172	172	361	0	0

Table 2 - Ratio of agreement between dominant shape and gold shape for the different grammars (columns) and different shape abstraction levels (rows).

Level	macrostate	microstate	OverDangle	NoDangle
**5**	0.810	0.837	0.844	0.789
**4**	0.673	0.687	0.687	0.667
**3**	0.667	0.680	0.687	0.660
**2**	0.639	0.646	0.660	0.633
**1**	0.544	0.531	0.578	0.571

Table 3 - Ratio of agreement between mfe shape and gold shape for the different grammars (columns) and different shape abstraction levels (rows).

Level	macrostate	microstate	OverDangle	NoDangle
**5**	0.844	0.844	0.878	0.796
**4**	0.714	0.714	0.728	0.673
**3**	0.701	0.701	0.728	0.667
**2**	0.667	0.667	0.687	0.633
**1**	0.585	0.585	0.612	0.585

Table 4 - Average rank of correct shape class.

Level	macrostate	microstate	OverDangle	NoDangle
**5**	1.6	1.6	1.6	1.7
**4**	2.3	2.6	2.8	2.6
**3**	2.7	3.0	3.0	3.0
**2**	14.3	54.8	54.8	48.3
**1**	186.5	90.0	75.0	66.5

Table 5 - Positions of correct shapes.

Level	macrostate				microstate				OverDangle				NoDangle			
	50%	75%	90%	100%	50%	75%	90%	100%	50%	75%	90%	100%	50%	75%	90%	100%
**5**	2	2	2	5	1	2	2	8	1	2	2	7	2	2	2	5
**4**	1	2	4	30	1	2	4	64	1	2	3	85	1	2	5	47
**3**	1	2	4	35	1	2	4	67	1	2	4	70	1	2	6	46
**2**	1	2	10	1027	1	2	12	6869	1	2	10	6697	1	3	24	5343
**1**	1	4	30	13492	2	4	25	9854	1	3	21	7758	1	4	41	5827

Table 6 - Relative runtime. The MacroState level 5 value equals 18.02 seconds on an Intel® Xeon® CPU L5420 @ 2.50GHz.

Level	macrostate	microstate	OverDangle	NoDangle
**5**	1.00	0.28	0.16	0.13
**4**	3.71	1.04	0.57	0.41
**3**	5.96	1.69	0.93	0.60
**2**	71.78	17.62	10.73	8.27
**1**	186.05	81.21	48.95	27.61

Table 7 - Relative memory. The MacroState level 5 value equals 30.2 MB resident set size.

Level	macrostate	microstate	OverDangle	NoDangle
**5**	1.00	0.27	0.25	0.22
**4**	4.23	0.80	0.83	0.53
**3**	6.00	1.22	1.19	0.68
**2**	46.08	8.70	8.78	7.65
**1**	105.72	28.63	50.27	23.78

Table 8 - Model similarity: shape probability shift

shape level 5

	ma	mi	Ov	No
ma	0.000	0.041	0.046	0.099
mi	0.041	0.000	0.042	0.132
Ov	0.046	0.042	0.000	0.126
No	0.099	0.132	0.126	0.000

shape level 4

	ma	mi	Ov	No
ma	0.000	0.045	0.054	0.112
mi	0.045	0.000	0.056	0.146
Ov	0.054	0.056	0.000	0.135
No	0.112	0.146	0.135	0.000

shape level 3

	ma	mi	Ov	No
ma	0.000	0.049	0.062	0.123
mi	0.049	0.000	0.068	0.159
Ov	0.062	0.068	0.000	0.139
No	0.123	0.159	0.139	0.000

shape level 2

	ma	mi	Ov	No
ma	0.000	0.050	0.063	0.126
mi	0.050	0.000	0.070	0.161
Ov	0.063	0.070	0.000	0.142
No	0.126	0.161	0.142	0.000

shape level 1

	ma	mi	Ov	No
ma	0.000	0.081	0.101	0.201
mi	0.082	0.000	0.131	0.254
Ov	0.086	0.120	0.000	0.194
No	0.185	0.243	0.194	0.000

ma = **ma**crostate
mi = **mi**crostate
Ov = **Ov**erDangle
No = **No**Dangle

Table 9 - Model similarity: average shape probability shift per shape

	ma	mi	Ov	No
ma	0.000	0.014	0.013	0.038
mi	0.013	0.000	0.014	0.047
Ov	0.011	0.013	0.000	0.040
No	0.045	0.061	0.056	0.000

shape level 5

	ma	mi	Ov	No
ma	0.000	0.009	0.005	0.022
mi	0.008	0.000	0.009	0.028
Ov	0.004	0.008	0.000	0.022
No	0.023	0.031	0.025	0.000

shape level 4

	ma	mi	Ov	No
ma	0.000	0.009	0.004	0.021
mi	0.008	0.000	0.009	0.027
Ov	0.003	0.009	0.000	0.020
No	0.021	0.029	0.023	0.000

shape level 3

	ma	mi	Ov	No
ma	0.000	0.006	0.001	0.014
mi	0.005	0.000	0.006	0.019
Ov	0.001	0.006	0.000	0.014
No	0.014	0.019	0.014	0.000

shape level 2

	ma	mi	Ov	No
ma	0.000	0.004	0.001	0.013
mi	0.005	0.000	0.005	0.017
Ov	0.001	0.005	0.000	0.013
No	0.013	0.017	0.013	0.000

shape level 1

ma = **ma**crostate
mi = **mi**crostate
Ov = **Ov**erDangle
No = **No**Dangle

2 FR3D:3A set

Table 10 - Comparison of different MFE prediction programs.

Using the 111 sequences from the FR3D:3A set, we repeated the same evaluation as shown the the Table 4 of the paper, but with *Turner 2004* energy parameters.

reference

prediction	1:pd	2:go	3:RN	4:no	5:UN	6:RN	7:ma	8:mi	9:UN	10:RN	11:ov
1: pdb structure	0	103									
2: gold structure	61	0									
3: RNAfold -d0	994	935	0	0	720	496	470	484	832	473	460
4: NoDangle	994	935	0	0	699	496	470	484	807	473	460
5: UNAfold -nodangle	907	854	716	691	0	696	662	692	679	694	675
6: RNAfold -d1	664	604	471	471	670	0	26	0	563	172	165
7: MacroState	634	576	450	450	637	28	0	27	529	145	137
8: MicroState	655	597	460	460	665	0	26	0	550	172	165
9: UNAfold	807	743	809	782	647	553	517	540	0	450	435
10: RNAfold -d2	720	658	474	474	689	197	167	197	484	0	0
11: OverDangle	704	643	463	463	676	190	160	190	471	0	0

Table 11 - Ratio of agreement between dominant shape and gold shape for the different grammars (columns) and different shape abstraction levels (rows).

Level	macrostate	microstate	OverDangle	NoDangle
**5**	0.820	0.847	0.802	0.649
**4**	0.658	0.685	0.622	0.523
**3**	0.559	0.595	0.550	0.450
**2**	0.568	0.568	0.550	0.459
**1**	0.387	0.396	0.405	0.360

Table 12 - Ratio of agreement between mfe shape and gold shape for the different grammars (columns) and different shape abstraction levels (rows).

Level	macrostate	microstate	OverDangle	NoDangle
**5**	0.847	0.847	0.847	0.694
**4**	0.676	0.676	0.667	0.541
**3**	0.595	0.595	0.586	0.468
**2**	0.586	0.586	0.568	0.459
**1**	0.423	0.432	0.423	0.369

Table 13 - Average rank of correct shape class.

Level	macrostate	microstate	OverDangle	NoDangle
**5**	1.4	1.4	1.4	1.7
**4**	1.8	1.7	1.9	2.8
**3**	2.6	2.6	2.5	3.4
**2**	6.1	5.5	6.4	11.5
**1**	35.7	11.7	16.6	29.3

Table 14 - Positions of correct shapes.

Level	macrostate				microstate				OverDangle				NoDangle			
	50%	75%	90%	100%	50%	75%	90%	100%	50%	75%	90%	100%	50%	75%	90%	100%
**5**	1	2	2	3	1	2	2	4	1	2	2	3	2	2	3	4
**4**	1	2	4	11	1	2	3	12	1	2	4	13	1	3	6	18
**3**	1	2	4	50	1	2	3	49	1	2	4	20	2	4	9	19
**2**	1	3	7	335	1	2	6	248	1	3	7	336	2	6	31	327
**1**	2	5	26	2714	2	4	17	472	2	6	24	611	3	16	84	547

Table 15 - Relative runtime. The MacroState level 5 value equals 15.05 seconds on an Intel® Xeon® CPU L5420 @ 2.50GHz.

Level	macrostate	microstate	OverDangle	NoDangle
**5**	1.00	0.30	0.17	0.15
**4**	2.71	0.82	0.47	0.36
**3**	4.10	1.22	0.70	0.50
**2**	34.84	8.93	5.93	4.55
**1**	82.18	34.20	23.61	12.95

Table 16 - Relative memory. The MacroState level 5 value equals 30.1 MB resident set size.

Level	macrostate	microstate	OverDangle	NoDangle
**5**	1.00	0.27	0.26	0.19
**4**	4.24	0.89	0.80	0.51
**3**	6.95	1.41	1.32	0.70
**2**	53.83	10.50	9.35	6.56
**1**	112.79	29.79	32.63	17.25

Table 17 - Model similarity: shape probability shift

	ma	mi	Ov	No
ma	0.000	0.065	0.054	0.188
mi	0.065	0.000	0.060	0.241
Ov	0.054	0.060	0.000	0.216
No	0.188	0.241	0.216	0.000

shape level 5

	ma	mi	Ov	No
ma	0.000	0.070	0.077	0.204
mi	0.070	0.000	0.086	0.257
Ov	0.077	0.087	0.000	0.222
No	0.203	0.256	0.221	0.000

shape level 4

	ma	mi	Ov	No
ma	0.000	0.074	0.091	0.224
mi	0.074	0.000	0.108	0.279
Ov	0.091	0.108	0.000	0.232
No	0.224	0.278	0.231	0.000

shape level 3

	ma	mi	Ov	No
ma	0.000	0.075	0.096	0.226
mi	0.075	0.000	0.114	0.281
Ov	0.096	0.114	0.000	0.233
No	0.226	0.280	0.232	0.000

shape level 2

	ma	mi	Ov	No
ma	0.000	0.120	0.179	0.310
mi	0.124	0.000	0.219	0.385
Ov	0.163	0.201	0.000	0.273
No	0.296	0.367	0.273	0.000

shape level 1

ma = **ma**crostate
mi = **mi**crostate
Ov = **Ov**erDangle
No = **No**Dangle

Table 18 - Model similarity: average shape probability shift per shape

shape level 5

	ma	mi	Ov	No
ma	0.000	0.020	0.015	0.057
mi	0.019	0.000	0.019	0.071
Ov	0.013	0.018	0.000	0.059
No	0.068	0.091	0.079	0.000

shape level 4

	ma	mi	Ov	No
ma	0.000	0.008	0.005	0.022
mi	0.008	0.000	0.009	0.029
Ov	0.005	0.008	0.000	0.022
No	0.024	0.032	0.026	0.000

shape level 3

	ma	mi	Ov	No
ma	0.000	0.007	0.005	0.022
mi	0.006	0.000	0.009	0.027
Ov	0.004	0.008	0.000	0.021
No	0.024	0.030	0.025	0.000

shape level 2

	ma	mi	Ov	No
ma	0.000	0.003	0.002	0.013
mi	0.003	0.000	0.004	0.016
Ov	0.002	0.004	0.000	0.013
No	0.013	0.015	0.013	0.000

shape level 1

	ma	mi	Ov	No
ma	0.000	0.004	0.002	0.011
mi	0.005	0.000	0.007	0.017
Ov	0.002	0.006	0.000	0.011
No	0.012	0.016	0.011	0.000

ma = **ma**crostate
mi = **mi**crostate
Ov = **Ov**erDangle
No = **No**Dangle

3 FR3D:4A

Table 19 - Comparison of different MFE prediction programs.

Using the 136 sequences from the FR3D:4A set, we repeated the same evaluation as shown the the Table 4 of the paper, but with *Turner 2004* energy parameters.

reference

prediction	1:pd	2:go	3:RN	4:no	5:UN	6:RN	7:ma	8:mi	9:UN	10:RN	11:ov
1: pdb structure	0	93									
2: gold structure	83	0									
3: RNAfold -d0	1396	1333	0	0	975	639	662	638	1095	725	724
4: NoDangle	1386	1323	0	0	922	605	628	604	1036	693	692
5: UNAfold -nodangle	1258	1187	978	918	0	896	842	894	856	869	853
6: RNAfold -d1	1002	935	612	575	867	0	69	0	730	376	367
7: MacroState	953	886	642	605	814	68	0	65	672	302	292
8: MicroState	998	931	612	575	864	0	66	0	725	368	359
9: UNAfold	1135	1071	1064	1002	809	710	654	707	0	583	579
10: RNAfold -d2	958	883	730	695	862	402	328	397	630	0	0
11: OverDangle	946	871	730	695	852	393	319	388	626	0	0

Table 20 - Ratio of agreement between dominant shape and gold shape for the different grammars (columns) and different shape abstraction levels (rows).

Level	macrostate	microstate	OverDangle	NoDangle
**5**	0.772	0.809	0.794	0.618
**4**	0.581	0.610	0.566	0.456
**3**	0.529	0.551	0.529	0.404
**2**	0.515	0.529	0.529	0.412
**1**	0.346	0.338	0.375	0.324

Table 21 - Ratio of agreement between mfe shape and gold shape for the different grammars (columns) and different shape abstraction levels (rows).

Level	macrostate	microstate	OverDangle	NoDangle
**5**	0.801	0.787	0.831	0.640
**4**	0.596	0.581	0.603	0.463
**3**	0.551	0.537	0.551	0.412
**2**	0.522	0.507	0.529	0.397
**1**	0.375	0.382	0.390	0.324

Table 22 - Average rank of correct shape class.

Level	macrostate	microstate	OverDangle	NoDangle
**5**	1.5	1.5	1.5	1.8
**4**	2.6	2.4	2.6	4.4
**3**	3.0	3.0	3.2	5.1
**2**	10.7	9.1	11.6	26.7
**1**	180.0	34.5	64.9	76.8

Table 23 - Positions of correct shapes.

Level	macrostate				microstate				OverDangle				NoDangle			
	50%	75%	90%	100%	50%	75%	90%	100%	50%	75%	90%	100%	50%	75%	90%	100%
**5**	1	2	2	7	1	2	2	6	1	2	2	6	2	2	3	9
**4**	1	3	4	29	1	2	4	25	1	2	4	34	2	4	7	140
**3**	1	3	4	48	1	2	4	56	1	2	5	51	2	5	9	151
**2**	1	3	14	240	1	3	11	231	1	3	11	269	2	11	44	1082
**1**	3	10	43	12095	2	7	37	2368	2	8	30	4663	3	35	149	3925

Table 24 - Relative runtime. The MacroState level 5 value equals 25.31 seconds on an Intel® Xeon® CPU L5420 @ 2.50GHz.

Level	macrostate	microstate	OverDangle	NoDangle
**5**	1.00	0.29	0.17	0.14
**4**	3.19	0.90	0.49	0.37
**3**	4.88	1.36	0.75	0.51
**2**	56.34	13.46	8.22	6.18
**1**	140.77	58.88	34.98	19.74

Table 25 - Relative memory. The MacroState level 5 value equals 30.1 MB resident set size.

**Level**	**macrostate**	**microstate**	**OverDangle**	**NoDangle**
**5**	1.00	0.27	0.26	0.21
**4**	4.23	0.89	0.80	0.50
**3**	6.94	1.41	1.32	0.69
**2**	55.82	10.48	9.34	6.55
**1**	112.60	29.74	51.57	18.42

Table 26 - Model similarity: shape probability shift

shape level 5

	ma	mi	Ov	No
ma	0.000	0.070	0.075	0.201
mi	0.070	0.000	0.069	0.260
Ov	0.075	0.069	0.000	0.248
No	0.201	0.260	0.248	0.000

shape level 4

	ma	mi	Ov	No
ma	0.000	0.074	0.097	0.216
mi	0.074	0.000	0.096	0.275
Ov	0.097	0.096	0.000	0.254
No	0.216	0.275	0.254	0.000

shape level 3

	ma	mi	Ov	No
ma	0.000	0.078	0.117	0.238
mi	0.079	0.000	0.123	0.299
Ov	0.117	0.123	0.000	0.260
No	0.238	0.298	0.259	0.000

shape level 2

	ma	mi	Ov	No
ma	0.000	0.080	0.122	0.241
mi	0.080	0.000	0.128	0.300
Ov	0.122	0.129	0.000	0.262
No	0.241	0.301	0.262	0.000

shape level 1

	ma	mi	Ov	No
ma	0.000	0.136	0.212	0.327
mi	0.139	0.000	0.242	0.402
Ov	0.189	0.222	0.000	0.300
No	0.305	0.382	0.300	0.000

ma = **ma**crostate
mi = **mi**crostate
Ov = **Ov**erDangle
No = **No**Dangle

Table 27 - Model similarity: average shape probability shift per shape

shape level 5

	ma	mi	Ov	No
ma	0.000	0.020	0.021	0.058
mi	0.019	0.000	0.019	0.071
Ov	0.018	0.019	0.000	0.063
No	0.070	0.093	0.088	0.000

shape level 4

	ma	mi	Ov	No
ma	0.000	0.007	0.006	0.018
mi	0.007	0.000	0.008	0.024
Ov	0.006	0.008	0.000	0.019
No	0.019	0.027	0.023	0.000

shape level 3

	ma	mi	Ov	No
ma	0.000	0.006	0.006	0.016
mi	0.006	0.000	0.008	0.020
Ov	0.005	0.008	0.000	0.015
No	0.017	0.022	0.019	0.000

shape level 2

	ma	mi	Ov	No
ma	0.000	0.003	0.002	0.006
mi	0.003	0.000	0.004	0.009
Ov	0.002	0.003	0.000	0.006
No	0.006	0.008	0.006	0.000

shape level 1

	ma	mi	Ov	No
ma	0.000	0.003	0.001	0.006
mi	0.004	0.000	0.005	0.010
Ov	0.001	0.004	0.000	0.006
No	0.007	0.010	0.006	0.000

ma = **ma**crostate
mi = **mi**crostate
Ov = **Ov**erDangle
No = **No**Dangle

4 RNAstrand:91

Table 28 - Comparison of different MFE prediction programs.

Using the 91 sequences from the rnastrand set, we repeated the same evaluation as shown the the Table 4 of the paper, but with *Turner 2004* energy parameters.

reference

prediction

	1:pd	2:go	3:RN	4:no	5:UN	6:RN	7:ma	8:mi	9:UN	10:RN	11:ov
1: pdb structure	0	0									
2: gold structure	0	0									
3: RNAfold -d0	558	558	0	0	386	217	247	217	399	245	245
4: NoDangle	522	522	0	0	355	186	216	186	368	214	214
5: UNAfold -nodangle	589	589	386	352	0	351	381	337	403	385	364
6: RNAfold -d1	448	448	220	186	353	0	31	0	239	128	107
7: MacroState	417	417	248	214	381	29	0	29	228	112	91
8: MicroState	446	446	220	186	337	0	29	0	221	124	103
9: UNAfold	530	530	386	352	387	221	210	204	0	213	199
10: RNAfold -d2	446	446	252	218	393	133	117	129	237	0	0
11: OverDangle	444	444	252	218	373	112	96	108	223	0	0
